# Multiomics Analysis Reveals Gut Virome–Bacteria–Metabolite Interactions and Their Associations with Symptoms in Patients with IBS-D

**DOI:** 10.3390/v16071054

**Published:** 2024-06-29

**Authors:** Peiwei Xie, Mei Luo, Jiahui Fan, Lishou Xiong

**Affiliations:** Department of Gastroenterology, The First Affiliated Hospital, Sun Yat-sen University, Guangzhou 510080, China

**Keywords:** diarrhea-predominant irritable bowel syndrome, virome, gut microbiota, multiomics, symptom, machine learning

## Abstract

The gut microbiota is involved in the pathogenesis of diarrhea-predominant irritable bowel syndrome (IBS-D), but few studies have focused on the role of the gut virome in IBS-D. We aimed to explore the characteristics of the gut virome in patients with IBS-D, its interactions with bacteria and metabolites, and the associations between gut multiomics profiles and symptoms. This study enrolled twelve patients with IBS-D and eight healthy controls (HCs). The stool samples were subjected to metavirome sequencing, 16S rRNA gene sequencing, and untargeted metabolomic analysis. The participants completed relevant scales to assess the severity of their gastrointestinal symptoms, depression, and anxiety. The results revealed unique DNA and RNA virome profiles in patients with IBS-D with significant alterations in the abundance of contigs from *Siphoviridae*, *Podoviridae*, *Microviridae*, *Picobirnaviridae*, and *Tombusviridae*. Single-omics co-occurrence network analyses demonstrated distinct differences in the gut virus, bacteria, and metabolite network patterns between patients with IBS-D and HCs. Multiomics networks revealed that short-chain fatty acid-producing bacteria occupied more core positions in IBS-D networks, but had fewer links to viruses. Amino acids and their derivatives exhibit unique connectivity patterns and centrality features within the IBS-D network. The gastrointestinal and psychological symptom factors of patients with IBS-D were highly clustered in the symptom–multiomics network compared with those of HCs. Machine learning models based on multiomics data can distinguish IBS-D patients from HCs and predict the scores of gastrointestinal and psychological symptoms. This study provides insights into the interactions among gut viruses, bacteria, metabolites, and clinical symptoms in patients with IBS-D, indicating further classification and personalized treatment for IBS-D.

## 1. Introduction

Irritable bowel syndrome (IBS) is one of the most common functional bowel diseases, affecting approximately 10% of the worldwide population [1]. It is classified into four subtypes: diarrhea-predominant (IBS-D), constipation-predominant (IBS-C), mixed bowel habits (IBS-M), and unclassified (IBS-U). IBS-D is the most prevalent subtype according to the Rome IV criteria, comprising 31% of all IBS cases [1]. In China, more than 70% of IBS patients are diagnosed with IBS-D based on the Rome II criteria [2]. Patients with IBS experience recurrent abdominal pain associated with changes in stool form or frequency, and one-third of them also suffer from anxiety or depression [3]. It is estimated that the annual health care expenditure for IBS patients in China is CNY 123 billion, accounting for 3.29% of the national health budget [4]. Despite its prevalence and impact, effective treatments for IBS remain limited, with current therapies improving symptoms in only one-third of patients [5]. IBS significantly impacts both individuals and society, presenting a substantial challenge for clinical management.

The pathogenesis of IBS is influenced by the gut microbiota, whose compositional and functional alterations can affect short-chain fatty acid (SCFA) production, mucosal barrier integrity, immune activation, and the brain–gut axis [6]. However, the characteristics of gut bacteria in IBS patients have been inconsistent across studies [7]. An important meta-analysis summarized the microbiota diversity and distinctive bacterial species in patients with IBS, but the results are highly heterogeneous [8]. Therapies manipulating the gut microbiota, such as fecal microbiota transplantation and probiotics, have shown promising efficacy on specific symptoms of IBS, but the mechanism of these therapies remains unclear [9,10]. Hence, there is no unified and recognized theory to explain how the gut microbiota participates in the development of IBS [7,11]. Notably, current studies on the gut microbiome in IBS patients have focused mainly on gut bacteria due to the widespread use of 16S rRNA sequencing; this leads to a knowledge gap regarding other critical gut microbiota components, such as viruses, fungi, and archaea. Further study of these “lost pearls” might deepen our understanding of the gut microbiota’s role in the onset of IBS.

Gut viruses, an essential component of the gut microbiota, are present in numbers that exceed those of gut bacteria by an order of magnitude [12]. The human gut virome includes eukaryotic viruses that primarily infect human cells and prokaryotic viruses (phages) that infect gut bacteria. Gut phages modulate gene expression and transfer of gut bacteria through lysis, lysogeny, pseudolysogeny, and bacterial budding. These processes affect bacterial metabolism, adhesion, and colonization behaviors, thereby regulating the structure and function of gut bacteria [13,14]. Gut viruses also influence the human immune system by modulating the release of cytokines, bacterial recognition, and immune cell activity [12]. Through these mechanisms, gut viruses and bacteria establish a tripartite consortium with the human host, collaboratively modulating human health [15].

Despite their indispensable ecological significance, studies on gut viruses in IBS patients are limited. Ansari et al. reported noticeable alterations in viral taxa and a significant reduction in the alpha diversity of the gut virome in IBS patients [16]. Another study from Coughlan et al. reported that the gut virome of individuals with IBS was more individual-specific than that of controls. Some viral clusters of *Mimiviridae*, *Podoviridae*, and *Siphoviridae* were significantly differentially abundant between the IBS and HC groups [17]. However, detailed information on IBS-D subtypes and RNA viruses is lacking. The interactions among gut viruses, bacteria, and IBS-D patients have not been fully elucidated. The role of gut viruses can only be better interpreted if they are integrated into a larger context of the gut microecosystem. Moreover, patients with IBS-D may present with different gastrointestinal, extragastrointestinal, and psychological symptoms [18]. Are the complex symptoms of IBS patients related to their equally complex gut viruses? Combined with multidimensional data on symptoms, gut viruses, bacteria, and metabolism in patients with IBS-D, we aimed to gain a system-level understanding of these issues.

In this study, we used metavirome analysis, multiomics analysis, and machine learning methods to explore the characteristics of the gut virome, its interactions with bacteria and metabolites, and the associations between gut multiomics profiles and the gastrointestinal and psychiatric symptoms of patients with IBS-D.

## 2. Materials and Methods

### 2.1. Study Cohorts

**Figure 1:**
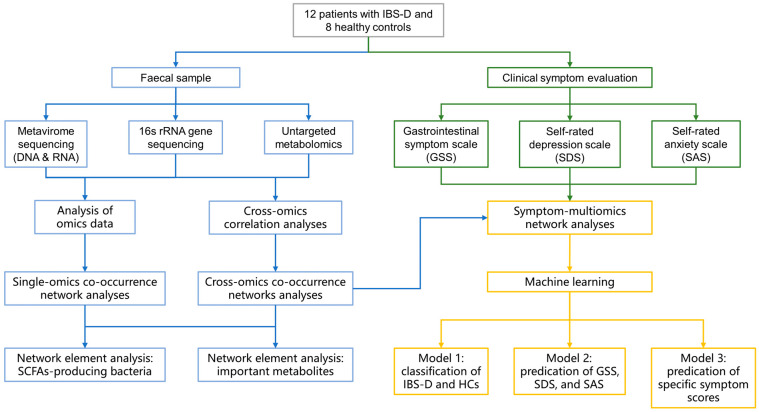
The flow chart of this study. IBS-D, diarrhea-predominant irritable bowel syndrome; SCFAs, short-chain fatty acids; GSS, Gastrointestinal Symptom Scale; SDS, Self-Rated Depression Scale; SAS, Self-Rated Anxiety Scale.

A flowchart of this study is illustrated in Figure 1. We recruited patients with IBS-D (*n* = 12) and healthy volunteers (*n* = 8) from the First Affiliated Hospital of Sun Yat-sen University. All the participants provided written informed consent. This study was approved by the Ethics Committee of the First Affiliated Hospital of Sun Yat-sen University (no. 2023482).

Participants aged 18–60 years were recruited for this study with no gender restrictions. Patients with IBS-D were diagnosed according to Rome IV criteria. In the healthy control group, participants had no past or present gastrointestinal symptoms. The exclusion criteria were as follows: 1. history of gastrointestinal surgery, 2. history of celiac disease, inflammatory bowel disease, lactose malabsorption, or other gastrointestinal diseases; 3. pregnant or lactating women; 4. oral use of antibiotics, probiotics, prebiotics, or other dietary supplements within three months; 5. use of nonsteroidal anti-inflammatory drugs, antianxiety drugs, and antidepressants within three months; or 6. dietary restrictions within six months, such as a low-fat diet or FODMAP.

All subjects completed the Gastrointestinal Symptom Scale (GSS), Self-Rated Depression Scale (SDS), and Self-Rated Anxiety Scale (SAS) [19,20]. All subjects provided fresh fecal samples that were stored in a −80 °C freezer within two hours after collection.

### 2.2. Metavirome Sequencing and Analysis

#### 2.2.1. Virus-like Particle (VLP) Enrichment and Metavirome Sequencing

Detailed protocols for VLP preparation and DNA and RNA extraction are available in the Supplementary Text S1. Five volumes of pre-cooled sterile Stabilization Buffer (SB) were added to the fecal samples. The mixture was ground, vortexed, subjected to freeze–thaw cycles with liquid nitrogen, and centrifuged to remove the pellet. Cellular debris was removed by filtration through a dual-layer membrane with pore sizes of 0.45 μm and 0.22 μm. The sample was ultracentrifuged at 4 °C and 160,000× *g* for 2 h to isolate the VLPs. For DNA and RNA extraction, the samples were processed by extracting viral nucleic acids using a viral extraction kit. Sequencing of the amplified libraries was performed using the PE150 protocol on the Illumina platform [21,22].

#### 2.2.2. Data Filtering and Assembly

We used Trimmomatic to evaluate the data quality and remove low-quality data [23]. After mapping the high-quality data with the host database (BWA, v0.7.17) [24], any clean reads that were shorter than 80% of the total read length were excluded. The assembly of clean data was conducted using Megahi (v1.1.2) [25]. The clean read utilization ratio was calculated using BWA (v0.7.17). The host sequence was eliminated by mapping with the contigs (blast, v2.9.0+). 

#### 2.2.3. Identification and Classification Annotation of Viral Sequences

First, we used CheckV to identify the potential virus sequence set in the assembly sequence [26]. The steps included the following: (1) predicting the previrus through identifying host contamination via the hidden Markov model (HMM); (2) detecting viral sequences through sequence matching (AAI) and HMM; (3) predicting the complete virus structure for the virus sequences obtained in step 2 by using CheckV to evaluate the existence of direct terminal repeats (DTRs) or inverted terminal repeats (ITRs) in sequences with high completeness (>90%), which required comparison with the reference virus terminal repeat sequence in the database and that the terminal repeat sequence had at least 20 bases; and (4) evaluating the quality of candidate virus sequences according to the sequence integrity and DTR/ITR characteristics. Virsorter2 was used to identify the virus sequence from the gene content and genomic structure characteristics and to screen sequences with high confidence as supplements to the CheckV results. Combining these two methods improved the sensitivity of virus identification.

#### 2.2.4. Viral Abundance Statistics and Diversity Analysis

Clean reads were compared to identified viral contigs. The reads per kilobase per million mapped reads (RPKM) values of each contig were calculated. We used BLAST (v2.9.0+) to compare the gene protein sequences with the virus sequences from the UniProtKB/Swiss-Prot database [27]. The threshold of the best hit was e < 0.001. Diamond was used to compare the sequences of nonredundant gene sets with the KEGG gene database for gene homology (e-value ≤ 0.001).

The alpha diversity was determined using the Shannon index, which was calculated based on the RPKM value of each sample. The beta diversity was determined using the Bray–Curtis distance matrix. Principal component analysis (PCA) and principal coordinate analysis (PCoA) were also conducted.

#### 2.2.5. Functional Analysis and Virus Host Prediction

We compared the protein sequences of identified viruses with the KEGG database and obtained functional annotation information. Phage host species information was predicted using two methods [28,29]. The CRISPR-spacer predicted phage–host relationships from scratch by identifying phage genomes that matched CRISPR spacers in genomic or metagenomic data. The viral protein families determined the species information, including domain, family, and genus, of the host according to the Baltimore classification of the protein sequence of the virus characteristic gene in the VPF database.

### 2.3. 16S rRNA Gene Sequencing

Detailed protocols for DNA concentration, PCR product extraction, and sequencing libraries are available in Supplementary Text S1. We extracted bacterial DNA using a MinkaGene stool DNA kit (MAGIGENE, Guangzhou, China) according to the manufacturer’s instructions. PCR products were extracted using a dedicated kit and sequenced on the Illumina MiSeq platform [30,31]. 

### 2.4. Untargeted Metabolomics Analysis

We performed LC–MS/MS analyses using a UHPLC system [32]. Then, the MS2 database was used for metabolite annotation [33]. Detailed protocols are available in Supplementary Text S1.

### 2.5. Co-Occurrence Network Construction and Topological Analyses

The co-occurrence networks were constructed based on Spearman correlation coefficients (|r| > 0.8, *p* < 0.001) of the gut virome, bacteria, and metabolites. The significance of pairwise correlations was determined after adjusting for the false discovery rate using the Benjamini–Hochberg procedure. The Spearman correlation coefficients between the three omics results were used to construct the cross-kingdom co-occurrence network. We retained the top 15% of the bacterial OTUs and the top 5% of the virome contigs to avoid false-positive correlations attributed to zero values. Gephi was used for network visualization and topological feature analyses [34]. The among- and within-module connectivity parameters (Pi and Zi) were calculated to analyze the topological roles of different nodes for the gut virome and bacteria, respectively. Nodes of short-chain fatty acid-producing bacteria and important metabolites were also selected for computing network topological features and visualization.

### 2.6. Machine Learning Model Training

Based on viral, bacterial, metabolic, and multiomics data, we used multiple deep learning methods to predict the IBS-D disease state, intestinal symptom factors, and psychological symptoms, respectively. The dataset was randomly divided into a training group, a validation group, and a test group. The respective percentages were 60%, 20% and 20%. Shapley Additive exPlanations (SHAP), random forest (RF), and L1-norm feature selection were used to select the top 20 significant features. First, we trained models to compare the ability of single-omics and multiomics data to classify IBS-D patients and HCs. The area under the curve (AUC), accuracy, precision, and specificity were used to evaluate the performance of the different models on the test set. We trained regression models to predict patients’ gastrointestinal and psychological symptoms, including overall GSS, SDS, and SAS scores and subgroup scores. Mean absolute error (MAE), mean absolute percentage error (MAPE), and R2 R^2^ index were used to evaluate the performance of the different regression models on the test set. The machine learning models include RF, support vector machine, logistic regression, decision tree (DT), Gaussian naive Bayes, multiple linear regression, least absolute shrinkage and selection operator (LASSO) regression, ridge regression, least squares boosting (LSBoost), extreme gradient boosting (XGBoost), Gaussian process regression, Gaussian kernel regression, multilayer perceptron (MLP) regression, extreme learning machine (ELM) regression, generalized regression neural network (GRNN), generalized additive model (GAM) regression, long short-term memory (LSTM) regression, gate recurrent unit (GRU) regression, convolutional neural network (CNN) regression, bi-directional LSTM (BiLSTM) Regression, and the combination method of the above models. The Deep Learning Toolbox and Statistics (version 14.6) and Machine Learning Toolbox (version 12.5) in MATLAB version 9.14.0 (R2023a) (The MathWorks Inc., Natick, MA, USA) were used to analyze and model data [35,36,37].

### 2.7. Statistical Analysis

T-tests or Mann–Whitney U tests were performed to detect differences in baseline characteristics depending on the normality of the data distribution. The Mann–Whitney U test was used to compare alpha diversity (Shannon index) and virus abundance between the two groups. Permutation multivariate analysis of variance test (PERMANOVA, Adonis test) was used to identify significant differences in microbial beta diversity. Linear discriminant analysis (LDA) effect size (LEfSe) analysis was used to identify differentially abundant viruses and bacteria between IBS-D and HC groups. Orthogonal partial least squares discriminant analysis (OPLS-DA) was used to identified differentially metabolites between IBS-D and HC groups. Spearman’s correlation analyses were performed to construct the co-occurrence network. Network topological features were analyzed using the Mann–Whitney U test. A *p*-value less than 0.05 was considered to indicate statistical significance. The statistical analyses were performed using R version 4.3.1.

## 3. Results

### 3.1. The Characteristics of the Gut Virome in IBS-D Patients

#### 3.1.1. Sequencing and Identification of the Gut Virome

Supplementary Table S1 summarizes the clinical characteristics of the participants (twelve patients with IBS-D and eight healthy controls (HCs)). 

DNA viruses: Sequencing of virus-like particles yielded an average of 27,018,667 reads per fecal sample after data quality control. The reads were assembled into 192,912 contigs with an average utilization rate of 99.27%. After virus identification by CheckV and Virsorter2, 31,220 virus contigs were identified, including 86.42% dsDNA viruses, 3.73% ssDNA viruses, and 9.8% unassigned viruses. More than 88.9% of contigs were identified as phages. Approximately 89.5% of the contigs were successfully classified at the phylum level but only 56.8% at the family level.

RNA viruses: After quality control, an average of 15,450,068 reads per fecal sample were obtained through sequencing. The assembly of reads resulted in 79,107 contigs, with an average utilization rate of 60.42%. BLAST (v2.9.0+) identified 2196 virus contigs, including 2091 phage contigs. Denovo virus sequence identification generated 12,086 virus contigs, including 11,257 contigs of phages. Finally, 648 RNA virus contigs were identified by combining these two methods. The proportion of RNA virus contigs among all the contigs was 5.52%.

#### 3.1.2. Alterations in the Composition of the Gut Virome in IBS-D Patients

The abundance statistics of the viruses were based on the RPKM values of the virus contig and the taxonomic annotations of the virus sequences. 

**Figure 2:**
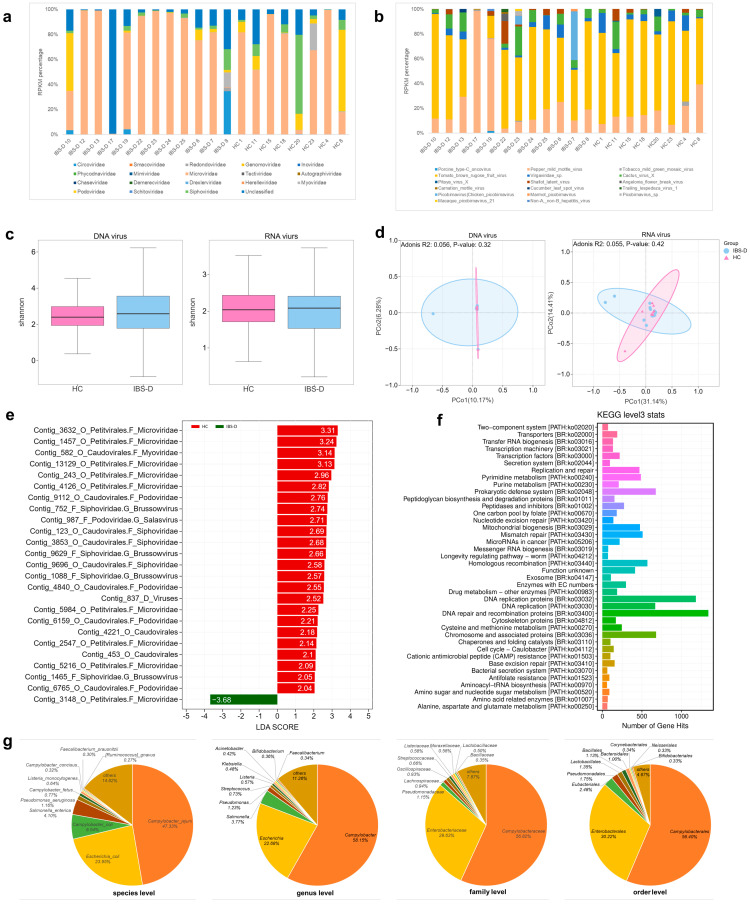
Gut virome characteristics of IBS-D and HC groups. (**a**) Barplot showing the abundance of gut DNA virus in IBS-D and HC at the family level. (**b**) Barplot showing the abundance of gut RNA virus in IBS-D and HC at the family level. (**c**) Boxplots of alpha diversity (Shannon) estimates for IBS and HC (DNA virus, *p* = 0.91; RNA virus, *p* = 0.73). For the boxplots, the boxes span from the first quartile to the third quartile, while the central line represents the median. (**d**) PCoA based on Bray–Curtis dissimilarity showed no significant differences in beta diversity between IBS-D and HC (DNA virus, Adonis test, *p* = 0.32; RNA virus, Adonis test, *p* = 0.42). (**e**) LEfSe analysis (*p* < 0.05, LDA score > 2) shows that the abundances of 25 contigs significantly differed between IBS-D and HC. (**f**) The enrichment pathways of IBS-D are based on KEGG level 3 annotation. (**g**) Bacteria host prediction of gut viruses in IBS-D at different taxonomic levels. IBS-D, diarrhea-predominant irritable bowel syndrome; HC, healthy control.

DNA viruses: The most abundant viruses in the IBS-D patients were *Microviridae* (70.59%), unclassified viruses (16.59%), *Podoviridae* (5.17%), *Circoviridae* (3.51%), and *Siphoviridae* (2.78%) at the family level (Figure 2a). The most abundant families in the HC group were *Microviridae* (62.1%), *Podoviridae* (12.6%), *Siphoviridae* (11.52%), unclassified viruses (10.69%), and *Myoviridae* (3%) (Figure 2a). The abundance of unclassified viruses or “viral dark matter” increased as the classification decreased to lower taxonomic levels. The abundances of unclassified viruses were 12.02%, 16.59%, 86.72%, and 99.24% at the order, family, genus, and species levels, respectively. This indicates that many gut viruses have not been classified yet. 

RNA viruses: The most abundant species in the IBS-D group were *Tomato brown rugose fruit virus* (52.39%), *Pepper mild mottle virus* (26.67%), *Cactus virus* X (7.33%), *Virgaviridae* sp. (3.81%), and *Chicken picobirnavirus* (3.77%) (Figure 2b). The most abundant species in the HC group were *Tomato brown rugose fruit virus* (56.98%), *Pepper mild mottle virus* (21.86%), *Virgaviridae* sp. (12.46%), *Cactus virus* X (2.16%), and *Shallot latent virus* (2.84%) (Figure 2b). Gut RNA viruses in the two groups were mainly derived from plant viruses. 

#### 3.1.3. Diversity of the Gut Virome in IBS-D Patients

DNA viruses: No significant differences in alpha diversity, as measured by Shannon index, were detected between the IBS-D and HC groups (Figure 2c, *p* = 0.91). PCoA based on Bray–Curtis dissimilarity revealed no significant differences in beta diversity between the HC and IBS-D groups (Figure 2d; Adonis test, *p* = 0.32).

RNA viruses: No significant differences in alpha diversity (Figure 2c, *p* = 0.73) or beta diversity (Figure 2d, Adonis test, *p* = 0.42) were observed between the two groups.

#### 3.1.4. Differences in the Abundances of Gut Viruses between the IBS-D and HCs Groups

DNA viruses: The abundance of 25 contigs differed significantly between the two groups (Figure 2e, LEfSe analysis, LDA score > 2, *p* < 0.05). Of those with increased abundance in HCs, seven were classified as *Siphoviridae* family (four as *Brussowvirus* genus), five were classified as *Podoviridae* family (one as *Salasvirus* genus), eight were classified as *Microviridae* family, one was classified as *Myoviridae* family, and the remaining three were unclassified at the family level (Figure 2e). Only one contig increased in IBS-D, which was classified as *Microviridae* family. Unclassified viruses were significantly increased in the IBS-D group when the taxonomic classification was moved to the species level (LEfSe analysis, *p* = 0.017) and order level (LEfSe analysis, *p* = 0.037), indicating that some viruses that have not been identified may also differ between the two groups. We also used the Mann–Whitney U test to identify viruses with significant differences between the two groups. At the genus level, *Cequinquevirus*, *Slopekvirus*, *Toutatisvirus*, and *Cepunavirus* were significantly depleted in the IBS-D group. However, *Pbunavirus* and *Oshimavirus* were significantly enriched in the patients with IBS-D (Supplementary Table S2). At the species level, *Klebsiella* virus Matisse, from the *Slopekvirus* genus, was significantly depleted in the IBS-D group (Supplementary Table S2).

RNA viruses: LEfSe analysis revealed that *Picobirnaviridae* (*p* = 0.04) and *Tombusviridae* (*p* = 0.04) were significantly more abundant in the IBS-D group. *Cycas necrotic stunt virus* (*p* = 0.025, LDA = 2.83) was significantly enriched in HCs (Supplementary Figure S1). According to the Mann–Whitney U test at the species level, *Pitaya virus* X (*p* = 0.04), *Cycas necrotic stunt virus* (*p* = 0.01), and *murine leukemia-related retroviruses* (*p* = 0.04) were significantly depleted in patients with IBS-D (Supplementary Table S3).

#### 3.1.5. Functional Annotation and Phage Host Prediction of the Gut Virome in IBS-D

DNA viruses: A total of 95,126 genes were predicted based on the virus contigs of IBS-D, of which 10,848 (11.4%) were mapped to the ViralZone database of UniProtKB/Swiss-Prot. The main enrichment pathways based on KEGG annotation were DNA repair and recombination proteins (ko03400), chromosome and associated proteins (ko03036), prokaryotic defense system (ko02048), DNA replication proteins (ko03032), DNA replication (ko03030), mismatch repair (ko03430), pyrimidine metabolism (ko00240), mitochondrial biogenesis (ko03029), purine metabolism (ko00230), amino acid related enzymes (ko01007), and alanine, aspartate and glutamate metabolism (ko00250) (Figure 2f).

RNA viruses: Based on the IBS-D RNA virus contigs, 331 genes were predicted, of which 247 (74.62%) were mapped to the ViralZone database. The main enrichment pathways based on KEGG annotation were hypothetical proteins and 2OG-Fe(II) oxygenase superfamily proteins.

According to the SpacePHARER host prediction results, 7266 contigs matched the bacterial host at the order level and 4050 contigs matched the bacterial host at the species level (Figure 2g). The top ten bacterial hosts in the IBS-D group were *Campylobacter jejuni*, *Escherichia coli*, *Campylobacter coli*, *Salmonella enterica*, *Pseudomonas aeruginosa*, *Campylobacter fetus*, *Listeria monocytogenes*, *Campylobacter concisus*, and *Faecalibacterium prausnitzii* at the species level. The top ten bacterial hosts at the genus level were *Campylobacter*, *Escherichia*, *Salmonella*, *Pseudomonas*, *Streptococcus*, *Listeria*, *Klebsiella*, *Acinetobacter*, *Bifidobacterium*, and *Faecalibacterium*.

### 3.2. Differences in Gut Bacteria between IBS-D Patients and HCs Were Associated with SCFA-Producing Bacteria and Amino Acid Functional Pathway Alterations 

A total of 3082 OTUs were assembled based on 2,001,882 16S rDNA sequences (100,094.10 ± 7213.95 per sample, mean ± SD). The two groups shared 1342 OTUs, of which 136 were observed in all samples. There were 1222 unique OTUs in the IBS-D group and 518 OTUs in the HCs.

**Figure 3:**
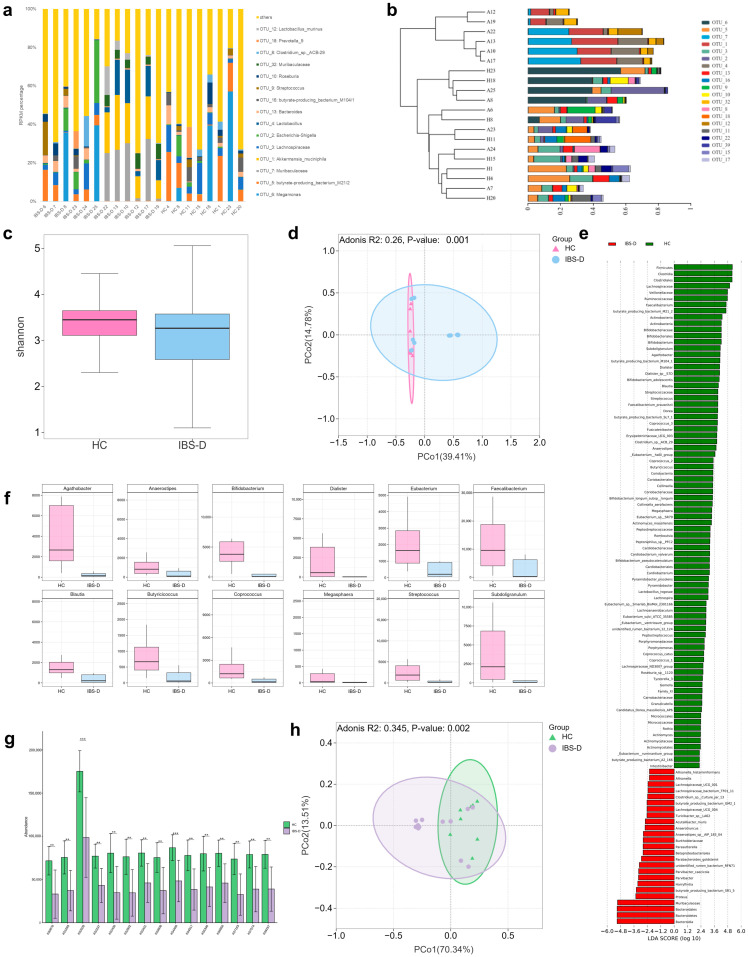
Gut bacteria characteristics of IBS-D and HC. (**a**) Barplot showing the abundance of gut bacteria in IBS-D and HC at the OTU level. (**b**) Phylogenetic tree of different samples at the OTU level. (**c**) Boxplots of alpha diversity (Shannon) estimates for IBS and HC (*p* = 0.53). For the boxplots, the boxes span from the first quartile to the third quartile, while the central line represents the median. (**d**) The bacterial community structure of IBS-D was significantly alternated compared to HC (Adonis test, *p* = 0.001). (**e**) LEfSe analysis identified 106 differentially abundant taxa between IBS-D and HC. (**f**) Boxplots show short-chain fatty acid-producing bacteria with significant differences in abundance between IBS-D and HC (*p* < 0.05). (**g**) Fifteen KEGG pathways significantly differed between IBS-D and HC (*p* < 0.05, ** *p* < 0.01, *** *p* < 0.001). (**h**) Adonis analysis based on Bray–Curtis dissimilarities also indicated significant alterations in KEGG functional pathways between IBS-D and HC (Adonis test, *p* = 0.002). IBS-D, diarrhea-predominant irritable bowel syndrome; HC, healthy control.

Figure 3a shows the gut bacterial compositions of the two groups at the OTU level. The dominant bacterial families of the IBS-D group were *Muribaculaceae* (22.3%), *Lachnospiraceae* (19.7%), *Akkermansiaceae* (10.91%), *Ruminococcaceae* (9.9%), *Lactobacillaceae* (9.6%), and *Veillonellaceae* (8.6%). The dominant bacterial families of the HC group were *Lachnospiraceae* (35.7%), *Ruminococcaceae* (20.2%), *Veillonellaceae* (16.6%), *Bacteroidaceae* (6.0%), *Bifidobacteriaceae* (4.6%), and *Enterobacteriaceae* (3.9%). Figure 3b shows the phylogenetic tree of different samples at the OTU level.

We did not observe significant differences in alpha diversity between the two groups (Figure 3c, *p* = 0.53). The Adonis test indicated that the bacterial community structure of the IBS-D patients was significantly altered compared to that of the HCs (Figure 3d, Adonis test, *p* = 0.001).

LEfSe analysis identified 106 taxa that were differentially abundant between the two groups, among which 25 were enriched in IBS-D patients and 81 were enriched in HCs (Figure 3e). Notably, the abundance of SCFA-producing bacteria differed between the two groups. The relative abundances of *Anaerostipies*, *Anaerotruncus*, *butyrate-producing bacterium* SR1-5, and GM2-1 were significantly enriched in the IBS-D group compared to those in the HCs. In contrast, the relative abundances of *Bifidobacterium*, *Streptococcus*, *Eubacterium*, *Agathobacter*, *Anaerostipes*, *Blautia*, *Coprococcus*, *Butyricicoccus*, *Faecalibacterium*, *Subdoligranulum*, *Dialister*, and *Megasphaera* were significantly enriched in the HC group (Figure 3f, *p* < 0.05). The abundance of some probiotics, including *Bifidobacterium adolescentis*, *Bifidobacterium longum* subsp. *longum*, and *Bifidobacterium pseudocatenulatum*, was also enriched in the HC group.

The PICRUSt and KEGG Orthology databases were used to conduct functional predictions for the two groups. The two groups shared 5798 KEGG pathways. In addition, 15 pathways were significantly different between the two groups (Figure 3g; *p* < 0.05). Pathway K02029 (polar amino acid transport system permease protein) was significantly enriched in the HC group. Adonis analysis based on Bray–Curtis dissimilarities also indicated significant alterations in the functional pathways between the two groups (Figure 3h, Adonis test, *p* = 0.002).

### 3.3. Alterations in Gut Metabolites in IBS-D Patients Were Correlated with Disordered Amino Acid Metabolism

A total of 16,523 metabolites in the fecal samples were obtained by an LC–MS/MS-based nontargeted metabolomics method, among which 724 metabolites were annotated in the databases. OPLS-DA analysis indicated that the metabolites were differentially abundant between the two groups (Supplementary Figures S2 and S3). Twenty-three annotated metabolites in the IBS-D group were differentially regulated (eleven upregulated and twelve downregulated) compared with those in the HC group (Figure S2a,b). KEGG enrichment analysis revealed that differentially abundant metabolites were associated with endocrine and amino acid metabolism (Figure S2c). Combining the results of the enrichment analysis and topology analysis, the following pathways were closely related to differentially abundant metabolites: hsa00260 (glycine, serine, and threonine metabolism), hsa00860 (porphyrin and chlorophyll metabolism), hsa00100 (steroid biosynthesis), and hsa00330 (arginine and proline metabolism) (Figure S2d).

### 3.4. Differential Gut Viruses Were Significantly Associated with Clinical Symptoms, Gut Bacteria, and Metabolites in IBS-D Patients

**Figure 4:**
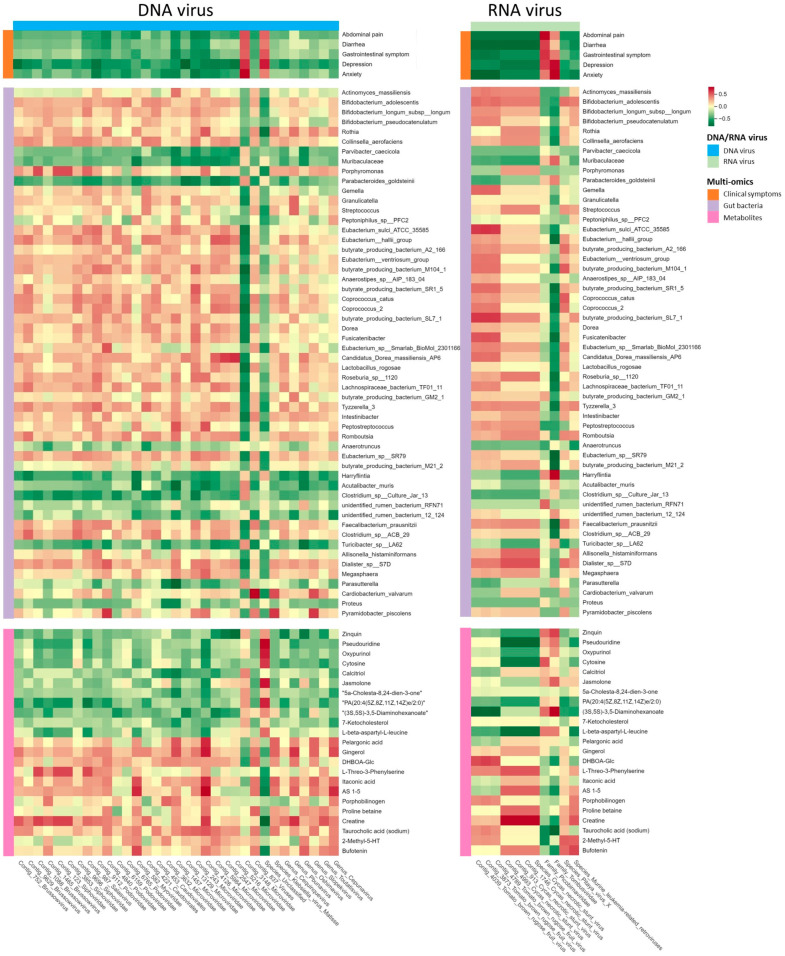
The heatmap shows color-coded Spearman’s correlations of differential DNA and RNA viruses (the horizontal bars) with clinical symptoms, gut bacteria, and metabolites (the longitudinal bars). Red indicates a positive correlation and green indicates a negative correlation. Colors of different multiomics data are shown in the legend.

We explored the cross-omics associations between gut viruses, clinical symptoms, gut bacteria, and metabolites in patients with IBS-D. Elements that differed significantly between the two groups were subjected to Spearman’s correlation analysis. Regarding the connection between viruses and symptoms, negative correlations were observed between the depression score and four *Microviridae* contigs, one *Brussowvirus* contig, and one *Podoviridae* contig (Figure 4; |r| > 0.6, *p* < 0.05). The anxiety score was negatively correlated with one *Microviridae* contig and one *Podoviridae* contig (Figure 4; |r| > 0.6, *p* < 0.05). For the virus–bacteria correlation, the differential gut viruses exhibited 58 significant correlations with the differential gut bacteria (Figure 4, |r| > 0.6, *p* < 0.05). Here, we focused on the association between gut viruses and SCFA-producing bacteria. Four *Microviridae* contigs were positively correlated with *butyrate-producing bacterium* M104-1, *Coprococcus* 2, *Eubacterium hallii* group, *Dialister* sp. S7D, *Megasphaera*, *Eubacterium hallii* group, *Coprococcus catus*, and *butyrate-producing bacterium* SR1-5. The two Podoviridae contigs were positively correlated with *the butyrate-producing bacteria* M104-1 and *Dialister* sp. S7D. *Coprococcus catus* was positively correlated with a *Siphoviridae* contig. For virus–metabolite correlations, *Pbunavirus* was positively correlated with creatine. The three *Microviridae* contigs were positively correlated with gingerol, itaconic acid, and pelargonic acid. Our results suggest that alterations in gut viruses in patients with IBS-D may be linked to gut bacteria, metabolites, and clinical symptoms.

### 3.5. Single-Omics Co-Occurrence Network Analyses of Gut Viruses, Bacteria, and Metabolites

In the previous section, the gut viruses of the IBS-D group established extensive associations with gut bacteria and metabolites; however, the heatmap in Figure 4 only illustrates the correlations between two specific elements in a 2-dimensional structure. We performed single-omics co-occurrence network analyses to further explore the network associations of gut viruses, bacteria, and metabolites. The co-occurrence network analyses of gut bacteria and metabolites are available in Supplementary Text S2.

**Figure 5:**
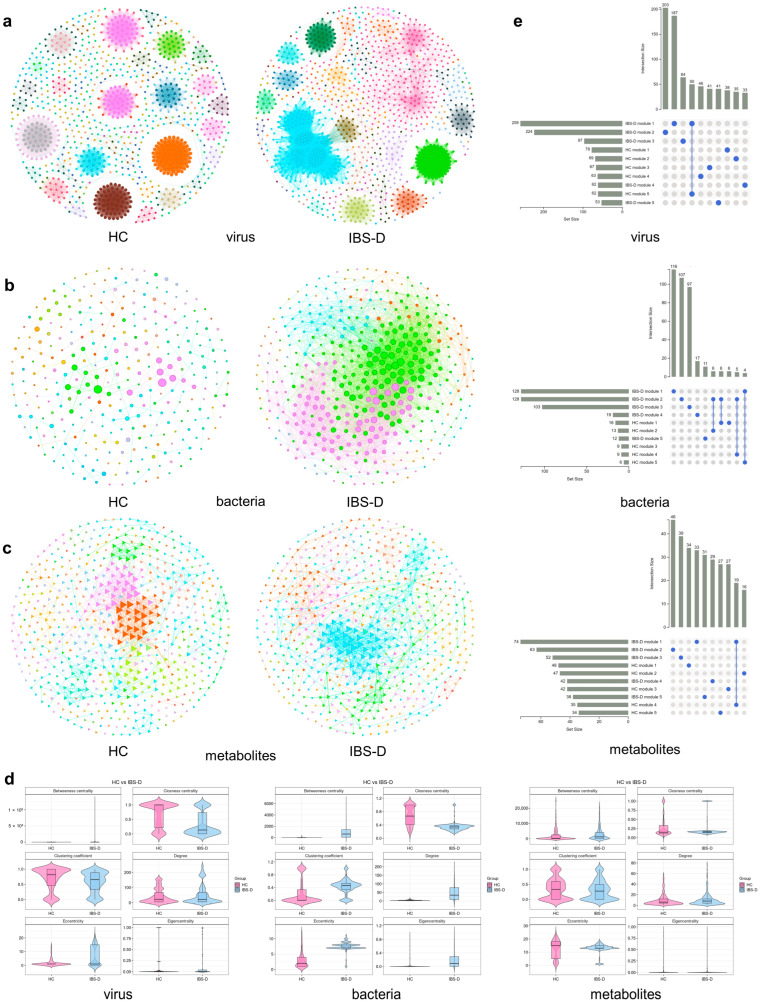
The single-omics co-occurrence network of gut virus, bacteria, and metabolites. (**a**) Gut virus co-occurrence networks in IBS-D and HC. Each hexagon node indicates a virus contig. The Spearman’s correlation analyses were used to construct the co-occurrence network (|r| > 0.8, *p* < 0.001). Node size is proportional to its degree. Edge thickness is proportional to Spearman’s rank correlation coefficient. Nodes and their edges are colored differently depending on the modularization result. (**b**) Gut bacteria co-occurrence networks in IBS-D and HC. Each circle node indicates a bacteria OTU. (**c**) Gut metabolites co-occurrence networks in IBS-D and HC. Each triangle node indicates a metabolite. (**d**) The violin plot shows the topological features of the co-occurrence networks in IBS-D and HC. (**e**) The UpSet plot illustrated low proportions of shared contigs in the top five modules of IBS-D and HC. The blue-filled dots connected by vertical lines indicate the intersection of the two modules. The vertical bars indicate the number of viral contigs within the intersections, and the horizontal bars indicate the total number of viral contigs in each module. IBS-D, diarrhea-predominant irritable bowel syndrome; HC, healthy control.

The virus co-occurrence network of IBS-D contained 1203 nodes and 31,369 edges, and that of HCs contained 1040 nodes and 22,778 edges (Figure 5a). The degrees of both networks were distributed according to power-law distributions, indicating scale-free features and nonrandom co-occurrence patterns (Supplementary Figure S4). Both networks had remarkably large positive edge proportions (>99.5%), implying dominance of coexistence between different viruses. Analysis of network topological features revealed that the IBS-D network had a higher average degree (26.76 vs. 21.9) but a lower modularity index (0.763 vs. 0.882) than the HC network. At the node level, the betweenness centrality of IBS-D was significantly higher than that of HCs, but the closeness centrality and clustering coefficient of IBS-D were significantly lower (Figure 5d, *p* < 0.001), suggesting that the interactions of gut viruses in patients with IBS-D might be more complex and decentralized than those in the HC network. High betweenness centrality values indicate that a node is relatively central to the network. We determined network hubs based on the top 20 betweenness centralities. The hub contigs of the IBS-D network were taxonomized into *Incheonvrus*, *Caudovirales*, *Duck circovirus*, unassigned virus, *Siphoviridae*, *Microviridae*, *Chlamydiamicrovirus*, and *Gyrovirus*. The hub contigs of the HC network were taxonomized into *Caudovirales*, *Siphoviridae*, *Incheonvrus*, *Microviridae*, and unclassified viruses. The Zi–Pi analyses indicated that there were 12 module hubs, 114 module connectors, and 1077 peripherals in the virus co-occurrence network of IBS-D, and the module hubs included *Podoviridae*, *Siphoviridae*, *Microviridae*, and *Brussowvirus* (Supplementary Figure S5). The HC network included 5 module hubs, 124 module connectors, and 912 peripherals, and the module hubs included *Chivirus*, *Brussowvirus*, *Siphoviridae*, and *Incheonvrus*. The IBS-D (0.763) and HC (0.882) networks had a modularity index above 0.4, indicating a high degree of modularity. The top 25% of the largest modules accounted for 76.6% of nodes in the IBS-D network. A total of 259 contigs comprised the largest module in the IBS-D network and were mainly assigned to *Siphoviridae* (32.4%), *Caudovirales* (20.1%), unassigned viruses (17.7%), *Myoviridae* (9.6%), and *Circoviridae* (6.9%). The largest module of the HC network consisted of 78 contigs, which were mainly assigned to *Myoviridae* (37.1%), *Caudovirales* (33.3%), *Siphoviridae* (21%), and unassigned viruses (7.8%). The top five modules in the two groups consisted of different contigs, and the proportion of shared contigs among them was low (Figure 5e, 0.01–16.49%). According to the above results, the IBS-D and HC networks differed in terms of four aspects: the basic network structure, topological characteristics, core nodes, and module composition. Our results demonstrated distinct differences in the virus co-occurrence network patterns between patients with IBS-D and HCs.

### 3.6. Multiomics Co-Occurrence Networks Integrating Gut Viruses, Bacteria, and Metabolites

**Figure 6:**
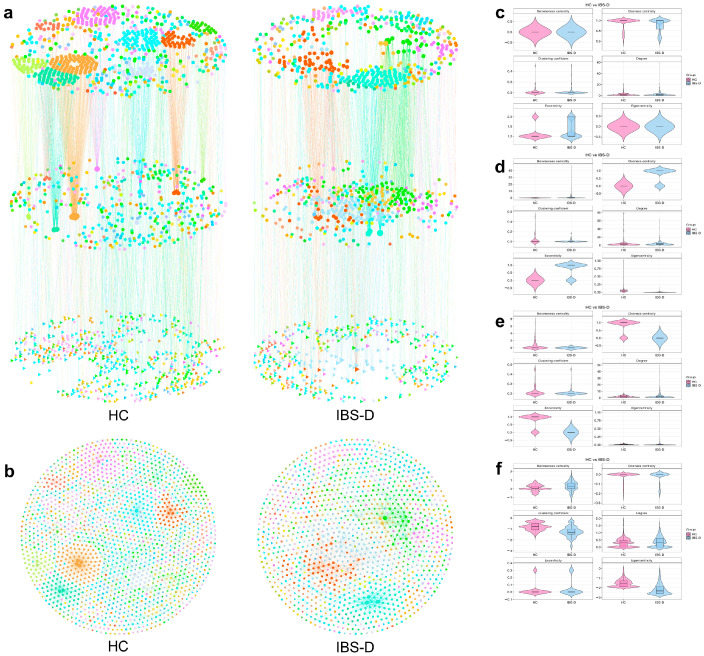
Multiomics co-occurrence network containing gut virus, bacteria, and metabolites. (**a**) Three-dimensional hierarchical view of the multiomics networks in IBS-D and HC. Cross-omics Spearman’s correlations constructed the network (|r| > 0.8, *p* < 0.001). The elements in the network diagram are divided into gut virus nodes (upper layer), gut bacteria nodes (middle layer), and gut metabolites nodes (lower layer). The hexagonal, circular, and triangular nodes indicate gut virus, bacteria, and metabolites. Node size is proportional to its degree. Edge thickness is proportional to Spearman’s rank correlation coefficient. Nodes and their edges are colored differently depending on the modularization result. (**b**) Two-dimensional plane plots of the multiomics networks in IBS-D and HC. Violin plots (**c**–**e**) show the subgroup topological features of gut viruses, bacteria, and metabolites in the multiomics networks, respectively. (**f**) The integral topological features of the multiomics networks. IBS-D, diarrhea-predominant irritable bowel syndrome; HCs, healthy controls.

Interomics Spearman correlations were used to construct the cross-omics co-occurrence network. The multiomics network of patients with IBS-D contained 1248 nodes, 779 bacteria–metabolite edges, 497 virus–metabolite edges, and 901 virus–bacteria edges (Figure 6a,b). The virus–bacterial edges consisted of 758 positive and 142 negative edges. The integral topological feature analyses showed that, compared to the HC network, the IBS-D network had a higher average degree (26.76 vs. 21.9) and betweenness centrality, but a lower closeness centrality, eigenvector centrality, and average clustering coefficient. This suggests that the cross-omics correlations in patients with IBS-D might be more intricate and dispersed than those in HCs. In subgroup topological feature analysis, the closeness centrality of virus nodes in IBS-D was lower than that in HCs (Figure 6c, *p* < 0.05). Compared to those in the HC network (Figure 6c), the bacterial nodes in the IBS-D group had a higher degree of betweenness centrality and closeness centrality but a lower clustering coefficient (Figure 6d, *p* < 0.05). The top 25% of the modules accounted for 69.9% of the nodes in the IBS-D network. The largest module of the IBS-D network mainly consisted of *Microviridae*, *Salasvirus*, *Mollicutes* RF39, *Ruminococcaceae* UCG-014, *Lachnospiraceae*, *Muribaculaceae*, Prostaglandin F1a, N-methyl-L-glutamic Acid and gamma-glutamylleucine. The largest module of the HC network consisted mainly of *Siphoviridae*, *Caudovirales*, *Microviridae*, *Ruminococcaceae* UCG-014, *Scardovia wiggsiae*, and *Bacteroides*. Overall, these results confirm the presence of a distinct virus–bacteria–metabolite phenotype in IBS-D patients compared to that in HCs.

### 3.7. SCFA-Producing Bacteria Occupied More Core Positions in Multiomics Networks but Had Fewer Links to Viruses

**Figure 7:**
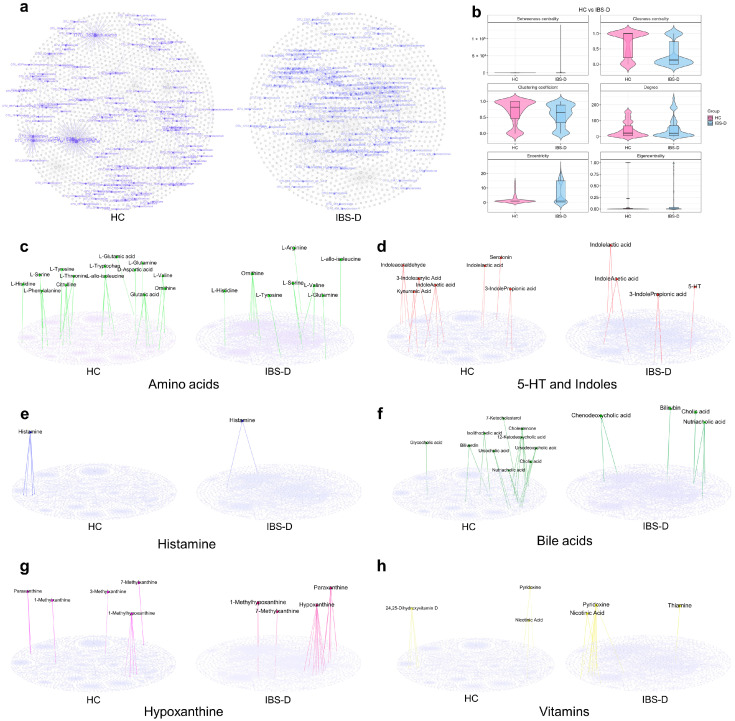
The short-chain fatty acid-producing bacteria and important metabolites in the multiomics co-occurrence network. (**a**) The SCFA-producing bacteria are highlighted with bright purple in the multiomics network. The rest of the elements in the network are gray. (**b**) The topological features of SCFA-producing bacteria in the multiomics networks. The network diagrams (**c**–**h**) show the important metabolites in the multiomics network. IBS-D, diarrhea-predominant irritable bowel syndrome; HC, healthy control.

SCFA-producing bacteria were selected from the bacterial and multiomics networks for topological feature analyses. For the bacterial co-occurrence networks, 118 OTUs from the IBS-D network and 95 OTUs from the HC network were assigned to the SCFA-producing bacteria. The SCFA-related bacteria in the IBS-D network had a higher average degree, clustering coefficient, and betweenness centrality but lower closeness centrality. In the multiomics co-occurrence network, 115 and 119 OTUs were assigned to SCFA-producing bacteria in the IBS-D and HC networks, respectively (Figure 7a). The SCFA-producing bacteria from the IBS-D network had a higher clustering coefficient, closeness centrality, and betweenness centrality but a lower eigenvector centrality (Figure 7b, *p* < 0.05). These results indicated that SCFA-producing bacteria in the IBS-D network were more often located in core positions than in HC. There were 169 edges between the viruses and SCFA-producing bacteria in the IBS-D network, which mainly included *Eubacterium*, *Faecalibacterium*, *Alistipes*, *Ruminococcus*, *Siphoviridae*, and *Caudovirales*. The multiomics network of HCs contained 329 edges between the SCFA-producing bacteria and viruses, which mainly included *Ruminococcus*, *Phascolarctobacterium*, *Dialister*, *Phascolarctobacterium*, *Microviridae*, *Podoviridae*, and *Siphoviridae*. Compared to those in the HC networks, SCFA-producing bacteria exhibited fewer co-occurring relationships with gut viruses in the IBS-D network.

### 3.8. Amino Acids and Their Derivatives Showed Unique Connectivity Patterns and Centrality Features in the Multiomics Network of IBS-D

Amino acids, 5-hydroxytryptamine (5-HT), indole derivatives, histamine, bile acids, vitamins, hypoxanthine, and related metabolites were selected to analyze their differences in the multiomics networks between the two groups.

The IBS-D network contained eight amino acids and fifteen related edges, which were mainly connected to *Ruminococcus* 2, *Ruminococcaceae* UCG-014, *Roseburia*, *Prevotella* 9, *Bacteroides*, *Lightbulbvirus*, *Myoviridae*, and *Microviridae* (Figure 7c). The HC network contained 14 amino acids and 28 related edges, which were mainly connected to *Haemophilus parainfluenzae*, *Desulfovibrio piger*, *Clostridiales bacterium* CIEAF 020, *Blautia*, *Parabacteroides*, *Bacteroides*, *Siphoviridae*, *Myoviridae*, and *Microviridae* (Figure 7c). The closeness centrality of the amino acids in the IBS-D network was significantly lower than that in the HC network. Tryptophan did not appear in the IBS-D multiomics network; however, there was a strong correlation between tryptophan and *Desulfovibrio* in the HC network (Figure 7c). 5-HT was correlated with *Chlamydiamicrovirus* and *Microviridae* in the IBS-D network, and Lachnospiraceae in the HC network (Figure 7d). The IBS-D network contained three indole derivatives and seven related edges, which were mainly connected to *Circoviridae*, *Microviridae*, and unassigned viruses (Figure 7d). The HC network contained three indole derivatives and thirteen related edges, which were mainly correlated with *Erysipelotrichaceae*, *Ruminococcaceae*, *Blautia* sp. *Marseille*-P3087, *Lachnospiraceae*, *Siphoviridae*, and *Microviridae*. Histamine was associated with *Lachnospiraceae* in the IBS-D group (Figure 7e). The network characteristics of bile acids, hypoxanthine, and vitamins are available in Supplementary Text S3. These results revealed distinct connectivity patterns and centrality differences in the essential metabolites between the two groups.

### 3.9. The Gastrointestinal and Psychological Symptom Factors of IBS-D Patients Were Highly Clustered in the Symptom-Multiomics Network

**Figure 8:**
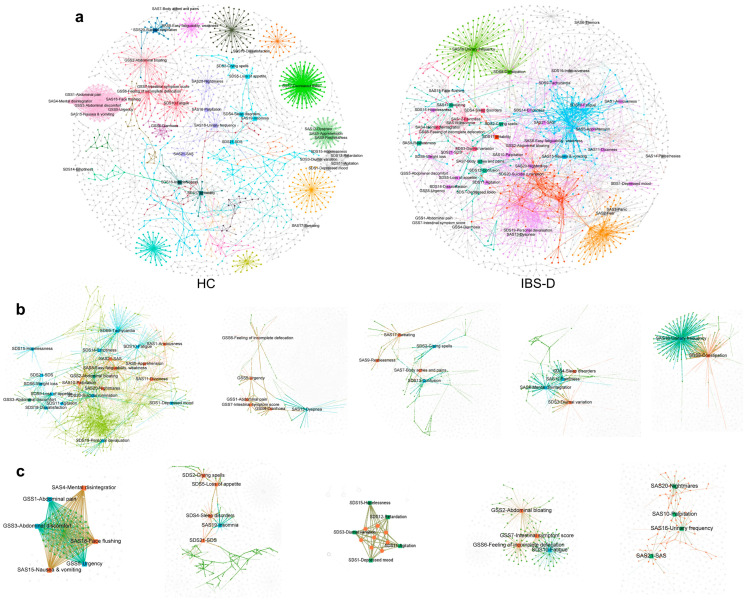
The gastrointestinal and psychological symptoms factors of IBS-D were more highly clustered in the symptom–multiomics network than that of HC. (**a**) Co-occurrence networks constructed from the symptom factors and multiomics data of IBS-D and HCs. Forty-eight GSS, SDS, and SAS symptom factors were added into the multiomics network based on the Spearman’s correlation between symptoms and multiomics data. Symptom factors, such as gut viruses, bacteria, and metabolites, are indicated by square, hexagonal, circular, and triangular nodes. Nodes and their edges are colored differently depending on the modularization result. (**b**) The top five modules in the symptom–multiomics network of IBS-D. The largest module (No. 387) accounts for 43.75% of all symptom factors. (**c**) The top five modules in the symptom–multiomics network of HC. The symptom factors are evenly distributed into different modules. IBS-D, diarrhea-predominant irritable bowel syndrome; HC, healthy control.

Patients with IBS-D may present with different gastrointestinal and psychological symptoms. We further analyzed the network correlations between the gut multiomics data and clinical symptoms of IBS-D. The three questionnaires completed by the participants were organized into a data matrix, including the GSS with seven subitems, the SDS score with 20 subitems, and the SAS score with 20 subitems. Then, Spearman correlation analyses were performed between the clinical symptom data and the gut virus, bacteria, and metabolite data. The 48 symptom factors of the symptom–multiomics network exhibited 896 correlations with the gut multiomics data of IBS-D patients, including 412 symptom–virus edges, 132 symptom–bacteria edges, and 352 symptom–metabolite edges (Figure 8a). The 48 symptom factors in the IBS-D network involved 14 modules, and the distribution of symptom factors in the modules showed a high concentration: the top three modules accounted for 66.67% of all symptom factors, and the largest module (No. 387) accounted for 43.75% of all symptom factors (Figure 8b and Supplementary Table S4). Module No. 387 involved two intestinal symptoms, twelve depressive symptoms, and seven anxious symptoms. The multiomics nodes of module No. 387 mainly included *Microviridae*, *Incheonvrus*, *Caudovirales*, *Ruminococcaceae* UCG-014, *Mollicutes* RF39, *Lachnospiraceae*, N-methyl-L-glutamic Acid, Hypoxanthine, and Glycylproline. The second largest module, No. 420, contained the most intestinal symptom factors, including abdominal pain, diarrhea, urgency, feeling of incomplete defecation, and total intestinal symptom score (Figure 8b). The multiomics nodes of module No. 420 mainly included *Microviridae*, *Lightbulbvirus*, *Andromedavirus*, *Brussowvirus*, *Scardovia wiggsiae*, *Bacteroides*, L-prolyl-L-proline, N-Ornithyl-L-taurine, and Tauroursodeoxycholic acid (Figure 8b). The HC network contained 7 GSS factors, 16 SDS factors, and 14 SAS factors, and was evenly distributed into 15 modules (Figure 8a and Supplementary Table S4). The top three modules accounted for 43.24% of all the symptom factors (Figure 8c). These results suggest that the symptom–multiomics network of IBS-D involves more psychological symptom factors. The symptom factors of IBS-D were highly clustered into several main modules in the network, which may participate in the pathogenesis of IBS-D.

### 3.10. Machine Learning Models Based on Multiomics Data Could Distinguish IBS-D Patients from HCs and Predict Gastrointestinal and Psychological Symptom Scores

**Figure 9:**
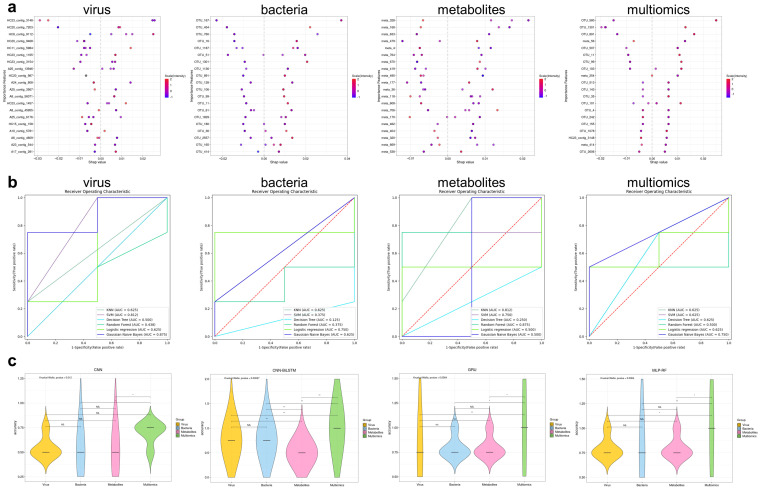
The prediction performances of different machine learning models for distinguishing IBS-D and HC. (**a**) The top 20 significant features selected by SHAP analyses in gut virus, bacteria, metabolite, and multiomics data. (**b**) ROC curves illustrate the potential of distinguishing IBS-D and HC by single-omics and multiomics data. (**c**) The prediction performances of different artificial neural networks in distinguishing IBS-D and HC. *, *p* < 0.05; **, *p* < 0.01; NS., Non-Statistical Significance. IBS-D, diarrhea-predominant irritable bowel syndrome; HC, healthy control.

To distinguish between patients with IBS-D and HCs, we first used six classical machine learning methods to model viruses, bacteria, metabolites, and multiomics data. Figure 9a shows the top 20 significant features selected by SHAP analyses for different datasets. The multiomics models consisted of one virus contig, sixteen bacterial OTUs, and three metabolites. The best-performing modeling methods in each database were GNB for gut viruses (AUC 0.875), logistic regression for gut bacteria (AUC 0.750), RF for gut metabolites (AUC 0.875), and GNB for multiomics data (AUC 0.750, Figure 9b). Given the high-dimensional characteristics of multiomics data, we used four artificial neural networks for disease-health classification modeling. The prediction accuracies of the four multiomics models based on CNN, CNN-BiLSTM, GRU, and MLP-RF were significantly higher than those of the other single-omics models (Figure 9c). The combination of the multiomics data significantly enhanced the accuracy of the model.

We predicted the total GSS, SDS, and SAS scores by training 34 different machine-learning regression models to evaluate global gastrointestinal and psychological conditions in patients with IBS-D. Compared to the single-omics prediction models for gastrointestinal symptoms (GSSs), the mean absolute error (MAE) of the multiomics model decreased to 1.35, the mean absolute percentage error (MAPE)decreased to 0.07, and the R2 increased to 0.85 (Table 1). For depression symptoms (SDS), the multiomics model achieved an MAE of 1.19, MAPE of 0.02, and R2 of 0.97 (Table 1). For anxiety symptoms (SAS), the multiomics model achieved an MAE of 0.85, MAPE of 0.02, and R2 of 0.89 (Table 1). The above results show that the multiomics ensemble can significantly improve model performance compared to the single-omics prediction model. 

Machine learning algorithms can also accurately predict the specific symptom scores of IBS-D. The core gastrointestinal symptoms of IBS-D include abdominal pain and diarrhea, which are critical diagnostic criteria for IBS-D. The SDS questionnaire also subdivided depressive symptoms into physiological concomitants (eight items) and psychological complications (ten items). The GAM regression model of the multiomics data demonstrated the best performance in predicting the abdominal pain score (MAE = 0.26, MAPE = 0.51, R2 = 0.92; Table 2). For the diarrhea score, the CNN-BiLSTM combination model of the multiomics data showed the best performance (MAE = 0.25, MAPE = 0.21, R2 = 0.97; Table 2). Regarding the physiological concomitants of depression, the ELM regression model of the multiomics data yielded a lower MAE of 0.52, a lower MAPE of 0.03, and a higher R2 of 0.96 compared with the single-omics models (Table 2). The CNN-LSTM-attention model of the multiomics data exhibited superior performance in predicting psychological concomitants of depression (MAE = 0.63, MAPE = 0.04, R2 = 0.98; Table 2).

These results suggest that, with appropriate machine learning methods, the gut multiomics model could distinguish IBS-D patients from HCs and accurately predict the severity of overall gastrointestinal and psychological symptoms, along with specific symptom factors, in IBS-D patients. Viruses, bacteria, and metabolites in specific modules may cause the corresponding clusters of symptoms.

## 4. Discussion

In this study, we integrated metavirome data, multiomics analysis, and machine learning methods to delineate the gut virome landscape, its interactions with bacteria and metabolites, and associations between gut multiomics profiles and symptoms in patients with IBS-D. Our results revealed distinct DNA and RNA gut virome compositions in patients with IBS-D, with notable variances in the abundance of contigs from *Siphoviridae*, *Podoviridae*, *Microviridae*, *Picobirnaviridae*, and *Tombusviridae*. Subsequent single-omics co-occurrence network analyses demonstrated distinct differences in the gut virus, bacterial, and metabolite network patterns between patients with IBS-D and HCs. Further analyses of multiomics co-occurrence networks revealed that SCFA-producing bacteria occupied more core positions in IBS-D networks but had fewer links to viruses. Functional analyses have linked shifts in the gut virome, bacteria, and metabolites to perturbations in amino acid metabolism, particularly in the glutamate, arginine, and proline pathways. These amino acids and their derivatives showed unique connectivity patterns and centrality features in the IBS-D multiomics network. Furthermore, the gastrointestinal and psychological symptom factors of patients with IBS-D were more highly clustered in the symptom-multiomics network than in the HCs. The machine learning model could distinguish IBS-D patients from HCs and predict the severity of overall gastrointestinal and psychological symptoms, along with the specific symptom scores of IBS-D patients. The incorporation of gut multiomics data significantly improved the prediction performance of the models.

To the best of our knowledge, Ansari et al. first characterized gut virus changes in 25 patients with IBS using metagenome sequencing and viral library alignment. The results showed that the diversity of *Megavirales* in patients with IBS-D differed from that in the HCs [16]. Coughlan et al. conducted metagenomic sequencing of fecal VLPs from 17 patients with IBS-D. The results indicated that the alpha diversity was significantly lower in the fecal viromes of IBS-D patients than in those of controls [17]. Based on the overall data of IBS patients, the above two pioneering studies also reported the dominant gut viruses in IBS patients and the viruses with significant differences in abundance. However, detailed results for the different IBS subtypes and RNA viruses are lacking. Our study focused on the gut viruses of patients with IBS-D, the subtype that affects the largest number of patients with IBS, and comprehensively reported changes in DNA and RNA viruses. Compared with those in HCs, the species and abundance proportions of the top five dominant gut viruses in IBS-D patients were different, whereas no significant differences in alpha or beta diversity were observed in either DNA or RNA viruses. In addition, we found that the abundance of *Brussowvirus* and *Salasvirus* significantly decreased in patients with IBS-D. The *Brussowvirus* genus, which includes many species that are temperate (pro)phages, could influence the biological characteristics of its host *Streptococcus thermophilus* [38,39]. For example, the integration of *Brussowvirus* 20617 within the host genome leads to compromised cell wall integrity, enhanced heat resistance, and increased surface adhesion [40]. The genus *Salasvirus* contains four species, namely, phi29, PZA, Goe6, and Gvx1, according to ICTV Taxonomy. The majority of these viruses infect *Bacillus subtilis*; however, they frequently target other closely related species as well [41]. *Salasvirus* phi29 is a typical phage of the *Salasvirus* that contains the smallest known dsDNA phage [42].

Regarding RNA viruses, our research indicated that *Picobirnaviridae* and *Tombusviridae* were significantly more abundant in patients with IBS-D. *Picobirnaviridae* belongs to a family of viruses with bisegmented dsRNA genomes. Several studies from China, India, and Brazil detected *Picobirnaviridae* in the stool of children with diarrhea [43,44,45,46]. Van Leeuwen et al. conducted diagnostic PCR tests on 83 stool samples from Dutch patients with unknown causes of diarrhea. The results revealed that 20% of the samples tested positive for genogroup I *Picobirnavirus* sequences [47]. The probability of detecting picornavirus in stool samples from immunocompromised patients was significantly increased, which supported the fact that *Picobirnaviridae* is an opportunistic pathogen that may cause diarrhea [48]. The excretion levels of *Picobirnaviridae* may be influenced by stress, the immune status of the host, and environmental conditions [49]. Although the role of PBVs as causative agents of intestinal disorders has not been established, there may be a link between *Picobirnaviridae* and IBS-D, which deserves further study.

Gut viruses and bacteria interact with their metabolites. A team from the United States conducted two studies on the use of bacteriophages for gastrointestinal health. The PHAGE study used a mixture of four bacteriophages targeting *Escherichia coli* to treat adults with gut symptoms. The treatments reduced the fecal *Escherichia coli* load and increased the abundance of members of the butyrate-producing genus *Eubacterium* [50]. The subsequent PHAGE-2 study showed a greater increase in *Lactobacillus* and SCFA-producing bacteria detected in the fecal sample of patients taking PreforPro with *B. lactis* than in those receiving the probiotic alone [51]. The *Bacteroides* phage BV01 decreases the ability of *Bacteroides vulgatus* to deconjugate bile acids by interrupting the transcription of the neighboring gene at attB [52]. In contrast, studies have shown that bacteria-derived metabolites can also affect viruses. SCFAs can activate the Ack pathway in *Lactobacillus reuteri*, leading to increased phage production through a RecA-dependent mechanism [53]. Scanlan indicated that the presence of bile salts had a negative impact on the abundance of phages as well as their adsorption efficiency in coevolving communities of *Escherichia coli* and the lytic phage PPO1 [54]. These studies explored how metabolites regulate ecological interactions between specific viruses and bacteria. Exploring virus–bacteria interactions involves the study of microscopic mechanisms and macroscopic analysis of the complex and elaborate interaction networks formed by the gut microbiota. Our analyses based on multiomics networks showed that SCFA-producing bacteria played more central roles in IBS-D networks, but had fewer links to viruses. In addition, compared to HCs, amino acids, 5-HT, indole derivatives, histamine, bile acids, vitamins, and hypoxanthine also exhibited different co-occurrence relationships in the IBS-D network. These results indicate that there is indeed a distinct network of viruses, bacteria, and metabolites in the gut of IBS-D patients compared to HCs, which could inform future studies on IBS-D pathogenesis.

Adding gastrointestinal and psychological symptom factors to the multiomics co-occurrence network for analysis is a highlight of our study. To our knowledge, this is the first study to correlate and visualize the complex cluster of IBS-D symptoms with multiomics data from the gut. The diagnosis, typing, and treatment of IBS are symptom oriented. Symptoms in patients with IBS-D include gastrointestinal, extraintestinal, and psychological symptoms. Given the complex clinical manifestations and unsatisfactory treatment efficacy of IBS, the academic community has found that a classification system based on stool form and defecation frequency cannot fully reflect the multidimensional nature of IBS, and researchers have attempted to classify IBS by adding psychological assessments [55]. Black et al. classified more than 1800 IBS patients into seven distinct subgroups using latent class analysis, which is characterized by varying degrees of gastrointestinal symptoms, extraintestinal symptoms, and psychological comorbidities [56]. The novel seven-cluster model indicated that quality of life, productivity, and social relationships were impaired significantly in the four groups bearing the greatest psychological burden [55]. Gut viruses, particularly bacteriophages, are closely associated with the brain’s cognitive, memory, and executive functions [57,58,59,60]. Therefore, we correlated the symptoms with the gut microbiota and metabolites and then used a modular approach to cluster the network elements. The results showed that the 48 symptom factors in the IBS-D network clustered into 14 modules. The largest module contained up to 40% of the symptoms and closely related multiomics elements. Four gastrointestinal symptoms were clustered in the second-largest module. We hypothesized that viruses, bacteria, and metabolites in specific modules may cause the corresponding clusters of symptoms. We used machine learning methods to fit the multiomics data. The results indicated that the gut multiomics model could distinguish IBS-D patients from HCs and accurately predict the severity of overall gastrointestinal and psychological symptoms and the specific symptom factors in IBS-D patients. This hypothesis was also supported by a large network meta-analysis that we previously performed, which showed that different probiotics had different effects on specific symptoms of IBS [61]. In summary, based on symptoms and gut microbiota patterns, it would be possible to develop precise microbial intervention therapies for the targeted and tailored treatment of IBS-D patients.

Our study had several strengths and limitations. We sequenced DNA and RNA viruses, whereas only one previous study reported gut RNA viruses from IBS-D patients [62]. Multiomics analyses including gut viruses, bacteria, and metabolites depict the gut microecosystem and its interactions with IBS-D patients in multiple dimensions. To our knowledge, this is the first study to correlate and visualize the intestinal and psychological symptoms of patients with IBS-D with gut multiomics data, including gut virus data. However, this study had a relatively small sample size, as it specifically targeted the subtype of diarrhea in patients with IBS. Our study could not fully evaluate the impact of gut viruses on other IBS subtypes; however, similar research ideas may be applied to IBS studies in other subtypes and regions. Our results contained a large amount of virus dark matter, as virus identification depends on the reference virus database. Studies have shown that up to 90% of the virus group sequences have little homology with the current reference database [63]. Given the typical challenges in multiomics integrations, including high dimensionality, sparsity, and multicollinearity, the biological significance of multiomics co-occurrence networks and machine learning prediction models remains to be clarified [18,64].

## 5. Conclusions

In this study, we revealed the characteristics of the gut virome in patients with IBS-D, its interactions with bacteria and metabolites, and the associations between gut multiomics profiles and gastrointestinal and psychological symptoms. We observed unique DNA and RNA virome profiles in patients with IBS-D. The multiomics networks of gut viruses, bacteria, and metabolites in patients with IBS-D showed different interactions and features than those in HCs, which mainly involved SCFA-producing bacteria and amino acids. Machine learning models based on multiomics data could distinguish IBS-D patients from HCs and predict gastrointestinal and psychological symptom scores. This study provides insights into the interactions between gut viruses, bacteria, metabolites, and clinical symptoms in patients with IBS-D, indicating the further classification and personalized treatment for IBS-D.

## Figures and Tables

**Table 1:** Summary of best performing machine learning models for GSS, SDS and SAS.

Scale	Performance Index	Virus	Bacteria	Metabolites	Multiomics
GSS	MAE	1.60	3.75	5.99	1.35
	MAPE	0.94	0.22	0.72	0.07
	R2	0.94	0.73	0.37	0.85
	ML algorithm	MLP-XGB	GRNN	GRU	SVM
SDS	MAE	3.03	7.73	1.67	1.19
	MAPE	0.08	0.15	0.04	0.02
	R2	0.71	0.15	0.88	0.97
	ML algorithm	BiLSTM-RF	RF	LSTM-XGB	LSTM
SAS	MAE	5.27	2.50	3.11	0.85
	MAPE	0.18	0.07	0.10	0.02
	R2	0.55	0.84	0.34	0.89
	ML algorithm	MLP	LSBoost	BiLSTM	CNN-GRU-SE

GSS, Gastrointestinal Symptom Scale; SDS, Self-Rated Depression Scale; SAS, Self-Rated Anxiety Scale; MAE, mean absolute error; MAPE, mean squared prediction error; R2, R-squared; ML, machine learning; MLP, multilayer perceptron; XGB, extreme gradient boosting; GRNN, generalized regression neural network; GRU, gate recurrent unit; SVM, support vector machine; BiLSTM, bi-directional long short-term memory; RF, random forest; LSTM, long short-term memory; LSBoost, least squares boosting; CNN-GRU-SE, convolutional neural network, gated recurrent unit, and self-attention.

**Table 2:** Summary of best performing machine learning models for specific symptoms of GSS and SDS.

Scale	Performance Index	Virus	Bacteria	Metabolites	Multiomics
GSS abdominal pain	MAE	0.70	0.45	0.58	0.26
	MAPE	0.42	0.53	0.13	0.51
	R2	0.70	0.64	0.87	0.92
	ML algorithm	LSBoost	GRNN	GRNN	GAM
GSS diarrhea	MAE	0.97	0.68	1.07	0.25
	MAPE	0.63	0.20	0.74	0.21
	R2	0.67	0.82	0.69	0.97
	ML algorithm	LSTM-XGB	MLP-SVM	MLP-RF	CNN-BiLSTM
SDS physiological concomitants	MAE	1.20	0.81	0.72	0.52
	MAPE	0.07	0.08	0.06	0.03
	R2	0.93	0.92	0.94	0.96
	ML algorithm	GAM	XGBoost	LSBoost	ELM
SDS psychological concomitants	MAE	2.05	3.09	0.92	0.63
	MAPE	0.09	0.14	0.07	0.04
	R2	0.69	0.53	0.97	0.98
	ML algorithm	CNN-LSTM-SE	SVM-RF	MLP-SVM	CNN-LSTM-SE

GSS, Gastrointestinal Symptom Scale; SDS, Self-Rated Depression Scale; MAE, mean absolute error; MAPE, mean squared prediction error; R2, R-squared; ML, machine learning; LSBoost, least squares boosting; GRNN, generalized regression neural network; GAM, generalized additive model; LSTM, long short-term memory; XGB, extreme gradient boosting; MLP, multilayer perceptron; SVM, support vector machine; RF, random forest; CNN, convolutional neural network; BiLSTM, bi-directional long short-term memory; ELM, extreme learning machine; CNN-LSTM-SE, convolutional neural network, gated recurrent unit, and self-attention.

## Data Availability

The raw data supporting the conclusions of this article will be made available by the authors on request.

## Supplementary Materials

The following supporting information can be downloaded at: https://www.mdpi.com/article/10.3390/v16071054/s1, Text S1. Supplemental materials and methods. Text S2. The co-occurrence network analyses of gut bacteria and metabolites. Text S3. The network characteristics of bile acids, hypoxanthine, and vitamins in multiomics networks. Table S1. Clinical characteristics of the study cohort. Table S2. DNA viruses with significant differences between the IBS-D and HC groups. Table S3. RNA viruses with significant differences between the IBS-D and HC groups (genus level). Table S4. The top five symptom modules in the symptom–multiomics networks. Figure S1. LEfSe analysis shows that the abundances of RNA virus significantly differed between IBS-D and HC groups (family levels). Figure S2. Gut metabolite characteristics of IBS-D and HC. Figure S3. Score scatter plot of OPLS-DA model for IBS-D vs. HC groups. Figure S4. Power-law distributions of single-omics co-occurrence networks. Figure S5. The Zi-Pi analyses.
