# Supplementary material for: Multiomics Analysis Reveals Gut Virome–Bacteria–Metabolite Interactions and Their Associations with Symptoms in Patients with IBS-D
Source: Viruses. 2024 Jun 29;16(7):1054. doi: 10.3390/v16071054 (PMC11281411; doi:10.3390/v16071054)

Supplementary material

Supplement to:

Multiomics analysis reveals gut virome-bacteria-metabolite interactions and their associations with symptoms in patients with IBS-D

Appendix

Text S1. Supplemental materials and methods. 2

Text S2. The co-occurrence network analyses of gut bacteria and metabolites. 4

Text S3. The network characteristics of bile acids, hypoxanthine, and vitamins in multi-omics networks. 5

Table S1. Clinical characteristics of the study cohort. 6

Table S2. DNA viruses with significant differences between the IBS-D and HC groups. 7

Table S3. RNA viruses with significant differences between the IBS-D and HC groups (genus level)...8

Table S4. The top 5 Symptoms modules in the symptom-multi-omics networks. 9

Figure S1. LEfSe analysis shows that the abundances of RNA virus significantly differed between IBS-D and HC groups (family levels). 11

Figure S2. Gut metabolites characteristics of IBS-D and HC. 12

Figure S3. Score scatter plot of OPLS-DA model for group IBS-D vs HC. 13

Figure S4. Power-law distributions of Single-omic co-occurrence network. 14

Figure S5. The Zi-Pi analyses. 15

Text S1 Supplemental materials and methods

Metavirome sequencing

Take 0.2 grams of the sample, grind it, and then add five volumes of precooled sterile Stabilization Buffer (SB). Vortex the mixture for 5 minutes, subject it to three rounds of liquid nitrogen freezing-thawing, and centrifuge at 12000 g for 5 minutes to remove the pellet. Eliminate cellular debris by filtering through a dual-layer membrane with pore sizes of 0.45μm and 0.22μm. Transfer the supernatant into an ultracentrifuge tube with 28% (w/w) sucrose using a syringe. Balance the tube carefully before centrifuging at 160000 g for 2 hours at 4°C using a Himac CP 100WX ultracentrifuge (Hitachi, Tokyo, Japan). After removing the supernatant, resuspend the pellet in 200 µL of SB buffer. Add EMB and EM in appropriate proportions and incubate the mixture at 37°C for 60 minutes. Then add Stop Solution (SS) in a 2µL ratio, mix thoroughly, and inactivate the enzyme reaction by incubating at 65~75°C for 10 minutes. Centrifuge the sample at 2000 rpm for 5 minutes, collect 200 µL of the supernatant, and store it at -20°C for future experiments.

For DNA extraction, samples are processed by extracting viral nucleic acids with a viral extraction kit, then undergoing whole-genome amplification utilizing the Illustra Ready-To-Go GenomiPhi V3 DNA Amplification Kit. To ensure quality, the amplified products are assessed using Thermo NanoDrop One, Life Technologies Qubit 4.0, and 1.5% agarose gel electrophoresis. During the RNA extraction process, viral RNA nucleic acids were extracted simultaneously using the Magen R6662-02 MagPure Viral RNA Mini LQ Kit. Following this, the Qiagen 150054 REPLI-g Cell WGA & WTA Kit was utilized to perform whole transcriptome amplification. The resulting amplifications underwent quality control checks with the Thermo NanoDrop One, Life Technologies Qubit 4.0, and 1.5% agarose gel electrophoresis. Qualified samples go through library construction with the ALFA-SEQ DNA Library Prep Kit or NEBNext® Ultra II ™ DNA Library Prep Kit (New England Biolabs, USA), following the provided guidelines. Sequencing of the amplified libraries is done using the PE150 protocol on either the Illumina platform.

16S rRNA gene sequencing

DNA integrity was measured using 1% agarose gel electrophoresis, and DNA concentration and purity were measured using a NanoDrop One spectrophotometer (Thermo Fisher Scientific, MA, United States). PCR was performed using a previously described method. A E.Z.N.A. Gel Extraction Kit was used to extract mixed PCR products. An NEBNext Ultra DNA Library Prep Kit (New England Biolabs, Massachusetts, USA) was used to generate sequencing libraries. Sequencing was conducted on the Illumina MiSeq platform. The original image data obtained by sequencing were converted into raw reads by base recognition analysis, and the results are stored in the FASTQ (fq) file format. Chimeras were removed from the raw reads by UCHIME. Sequences with more than 97% similarity were clustered into the same operational taxonomic units (OTUs). The relative abundance, alpha diversity, and beta diversity were calculated based on the OTU table.

Untargeted metabolomics analysis

The fecal samples (200 mg) were mixed with beads and 500 µL of extraction solution (MeOH:ACN: H2O, 2:2:1 (v/v)) containing deuterated internal standards. After vortexing for 30 s, the mixed solution was homogenized (35 Hz,4 min) and sonicated for 5 min in a 4 ℃ water bath, and the process was repeated three times. The samples were incubated for 1 h at -40 ℃ to precipitate proteins and then centrifuged at 12000 rpm (RCF=13800(×g), R= 8.6cm, 15 min,4 ℃). The quality

control (QC) sample was prepared by mixing an equal aliquot of the supernatant of the samples. We performed LC–MS/MS analyses by using a UHPLC system (Vanquish, Thermo Fisher Scientific) with a Waters ACQUITY UPLC BEH Amide coupled to an Orbitrap Exploris 120 mass spectrometer (Orbitrap MS, Thermo). The mobile phase consisted of ammonium acetate and ammonia hydroxide in water（both 25 mmol/L, pH = 9.75）(solvent A) and acetonitrile (solvent B), with the an autosampler temperature of 4 ℃ and an injection volume of 2 μL. The Orbitrap Exploris 120 mass spectrometer acquired MS/MS spectra in information-dependent acquisition (IDA) mode based on the acquisition software (Xcalibur, Thermo). The ESI source conditions were set as follows: sheath gas flow rate of 50 Arb, aux gas flow rate of 15 Arb, capillary temperature of 320 ℃, full MS resolution of 60000, MS/MS resolution of 15000, collision energy of SNCE 20/30/40, and spray voltage of 3.8 kV or -3.4 kV. The raw data were converted to the mzXML format using ProteoWizard and processed with R for peak detection, extraction, alignment, and integration. Then, the MS2 database was used for metabolite annotation. The cutoff for annotation was set at 0.3.

Text S2 The co-occurrence network analyses of gut bacteria and metabolites

The bacterial co-occurrence network analyses

The bacterial co-occurrence network of IBS-D patients contained 431 nodes and 10732 edges, and the presence of positive edges (73.5%) was more dominant than that of negative edges (26.5%) (Figure 6b). The health control network included 320 nodes and 584 edges. Topological feature analyses revealed that the IBS-D network had a higher average degree and betweenness centrality than the HC network did. However, the modularity index (0.992 vs. 1.396) and closeness centrality of patients with IBS-D were significantly lower than those of HCs (Figure 6d, $p < 0.05$). Similar to virus co-occurrence networks, the bacterial networks of patients with IBS-D were more connected and less modular than the HC network. Based on the top ten betweenness centralities, the IBS-D network shared three hubs (*Lachnospiraceae*, *Roseburia*, and *butyrate-producing bacterium* M104/1) with the HC network. The unique hub contigs of IBS-D were taxonomized into *Actinomyces*, *Fusobacterium*, *Ventriosum*, *Bacteroides*, and *Clostridium* sp. ACB-29. The unique hub contigs of HC included *Ruminococcaceae* UCG-014, *Oscilibacter massiliensis*, *Muribaculaceae*, and *Prevotellaceae*. Zi–Pi analyses revealed 1 module hub (Bacteroides), 67 module connectors, and 363 peripherals in the virus co-occurrence network of IBS-D patients. (Supplementary Figure S5). The HC network includes 93 module connectors and 227 peripherals. The bacterial co-occurrence network showed more intermodule communication than the viral co-occurrence network. The modularity indices of the IBS-D (0.992) and HC (1.396) networks were above 0.4, suggesting the presence of modularly structured networks. Compared to the HCs, the IBS-D group had fewer modules, but these had larger sizes. The four largest modules accounted for 88.11% of the nodes in the IBS-D network. The network structures of the gut bacterial communities in the IBS-D group were also altered.

The metabolite co-occurrence network analyses

The metabolite co-occurrence network of patients with IBS-D contained 595 nodes and 3103 edges, and the network of healthy controls included 605 nodes and 2944 edges (Figure 6c). Topological feature analysis revealed that the IBS-D network had a higher average degree, betweenness centrality, and eigenvector centrality ($p<0.001$). However, the average clustering coefficient of IBS-D patients was significantly lower than that of HCs (Figure 6d, $p < 0.05$). The metabolite networks of IBS-D were more evenly distributed than those of the HCs. Verimol F, Ac-DL-Trp-OH, Anserine, and L-Serine were indicated as the hub metabolites of IBS-D included. The hub metabolites of HCs included 2-ethylacrylic acid, D-pantothenoyl-L-cysteine, NAD, 1-minopropan-2-ol, and Nicotinic Acid. Zi–Pi analyses indicated that there were seven module hubs, 165 module connectors, and 423 peripherals in the virus co-occurrence network of IBS-D, and the module hubs included succinic acid, DL-methionine sulfoxide, Racemethionine, Pyridoxal, L-Methionine, NAD, and Norfloxacin (Supplementary Figure S5). The HC network included 4 module hubs, 160 module connectors, and 441 peripherals, and the module hubs included oxytetracycline, suberic acid, and 1-methylxanthine. These results demonstrated that the metabolite network patterns of the IBS-D group differred from those of the HCs.

Text S3 The network characteristics of bile acids, hypoxanthine, and vitamins in multi-omics networks.

Bile acids: The IBS-D network contain 4 bile acids and 9 related edges, which mainly connected with Tyzzerella_3, Siphoviridae, Caudovirales, and Circovirus. The HC network contain 10 bile acids and 26 related edges, which mainly connected with Megamonas, Ruminococcus, Lachnospiraceae, Bifidobacterium, Siphoviridae, Caudovirales, and Microviridae. The closeness centrality of bile acids in IBS-D network were significantly lower than that of HC.

Hypoxanthine: The IBS-D network contain 4 Hypoxanthine derivatives and 19 related edges, which mainly connected with Faecalibaculum, Lachnospiraceae, Eubacterium, Andromedavirus, Siphoviridae, Microviridae, and unassigned virus. The HC network contain 5 Hypoxanthine derivatives and 9 related edges, which mainly connected with Subdoligranulum, Enterobacteriaceae, Romboutsia, Blautia, Bacteroides, and Siphoviridae. The closeness centrality of bile acids in IBS-D network were significantly lower than that of HC.

Vitamins: The IBS-D network contain 3 Vitamins and 16 related edges, which mainly connected with Ruminococcaceae_UCG-014, Lachnospiraceae, Siphoviridae, Myoviridae, Caudovirales, and unassigned virus. The HC network contain 3 Vitamins and 7 related edges, which mainly connected with Enterobacteriaceae, Faecalibacterium, Lachnospiraceae, Bacteroides, and Bifidobacterium.

Table S1. Clinical characteristics of the study cohort.

Variables±	IBS-D	HC	*p* value
Age, years (mean±SD)	29.17±6.71	27±3.5	0.49
Female, n (%)	6 (50%)	3 (37.5%)	0.67
Body mass index, kg/m^2 (mean±SD)	20.65±2.78	21.35±1.08	0.5
Gastrointestinal symptom score	19.67±5.69	1.75±3.41	< 0.001
Abdominal pain	3.5±1.56	0.12±0.35	< 0.001
Distension	1.66±1.49	0.37±0.51	0.03
Abdominal discomfort	3.08±1.72	0.12±0.35	< 0.001
Diarrhea	4.17±1.26	0.5±1.07	< 0.001
Urgency	4.17±1.02	0.37±1.06	< 0.001
Incomplete defecation	3.08±1.72	0.25±0.46	< 0.001
Self-rating depressioin scale	53.12±9.42	32.18±5.29	< 0.001
Self-rating anxiety scale	43.43±3.88	43.12±5.51	< 0.001

Values are presented as mean±standard deviation for continuous variables or number (percentage) for categorical variables. Continuous variables were compared using the Student's t-test for normally distributed variables or Mann-Whitney U test for non-normally distributed variables. Categorical variables were compared using the Chi-square test or Fisher's exact test. IBS-D, diarrhea-predominant irritable bowel syndrome; HC, Healthy controls; SD, standard deviation.

Table S2. DNA viruses with significant differences between the IBS-D and HC groups

Taxon	Taxonomic level	IBS-D_average	HC_average	Difference	p-value*
Cequinquevirus	Genus level	0.292	3.066	2.774	0.036
Pbunavirus	Genus level	0.648	0.441	-0.207	0.047
Oshimavirus	Genus level	0.551	0.143	-0.409	0.050
Slopekvirus	Genus level	0.000	0.869	0.869	0.044
Toutatisvirus	Genus level	0.019	0.665	0.646	0.031
Cepunavirus	Genus level	0.000	0.498	0.498	0.015
Klebsiella_virus_Matisse	Species level	0	0.869	0.869	0.044

* Mann-Whitney U test

Table S3. RNA viruses with significant differences between the IBS-D and HC groups (genus level)

Taxon	Species	IBS-D average	HC average	Difference	p-value*
Pitaya_virus_X	Species	0.000	8497.783	8497.783	0.044
Cycas_necrotic_stunt_virus	Species	0.000	754.793	754.793	0.015
Murine_leukemia-related_retroviruses	Species	0.000	13.315	13.315	0.044

* Mann-Whitney U test

Table S4. The top 5 Symptoms modules in the symptom-multi-omics networks

Modules	Symptoms
IBS-D modules No.387	GSS2-Abdominal bloating<br>GSS3-Abdominal discomfort<br>SAS1-Anxiousness<br>SAS10-Palpitation<br>SAS11-Dizziness<br>SAS20-Nightmares<br>SAS21-SAS<br>SAS5-Apprehension<br>SAS8-Easy fatiguability, weakness<br>SDS1-Depressed mood<br>SDS10-Fatigue<br>SDS11-Agitation<br>SDS14-Emptiness<br>SDS15-Hopelessness<br>SDS18-Dissatisfaction<br>SDS19-Personal devaluation<br>SDS20-Suicidal rumination<br>SDS21-SDS<br>SDS5-Loss of appetite<br>SDS6-Weight loss<br>SDS9-Tachycardia
IBS-D modules No.420	GSS1-Abdominal pain<br>GSS4-Diarrhoea<br>GSS5-Urgency<br>GSS6-Feeling of incomplete defecation<br>GSS7-Intestinal symptom score<br>SAS13-Dyspnea
IBS-D modules No.401	SAS17-Sweating<br>SAS7-Body aches and pains<br>SAS9-Restlessness<br>SDS13-Confusion<br>SDS2-Crying spells
IBS-D modules No.384	SAS12-Faintness<br>SAS4-Mental disintegratior<br>SDS3-Diurnal variation<br>SDS4-Sleep disorders
IBS-D modules No.377	SAS16-Urinary frequency<br>SDS8-Constipation
HC modules No.433	GSS1-Abdominal pain<br>GSS3-Abdominal discomfort<br>GSS5-Urgency<br>SAS15-Nausea & vomiting

	SAS18-Face flushing<br>SAS4-Mental disintegratior
HC modules No.429	SAS19-Insomnia<br>SDS2-Crying spells<br>SDS21-SDS<br>SDS4-Sleep disorders<br>SDS5-Loss of appetite
HC modules No.442	SDS1-Depressed mood<br>SDS11-Agitation<br>SDS12-Retardation<br>SDS15-Hopelessness<br>SDS3-Diurnal variation
HC modules No.440	GSS2-Abdominal bloating<br>GSS6-Feeling of incomplete defecation<br>GSS7-Intestinal symptom score<br>SDS10-Fatigue
HC modules No.435	SAS10-Palpitation<br>SAS16-Urinary frequency<br>SAS20-Nightmares<br>SAS21-SAS

Figure S1. LEfSe analysis ($p < 0.05$, LDA score > 2) shows that the abundances of RNA virus significantly differed between IBS-D and HC groups (family levels).

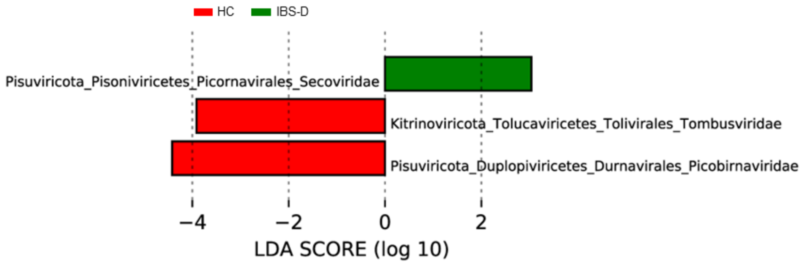

Figure S2 Gut metabolites characteristics of IBS-D and HC.

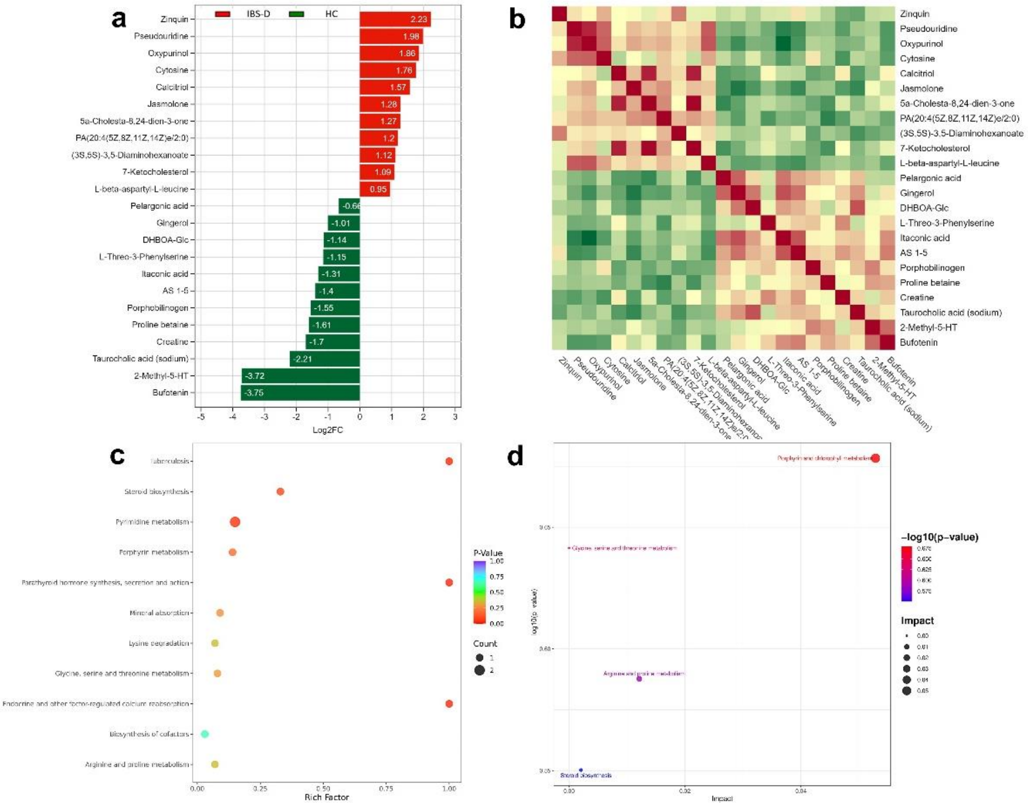

(a) Orthogonal projections to latent structures discriminant analysis (OPLS-DA) analysis identified 23 differential metabolites between IBS-D and HC. FC, fold change (VIP > 1, p < 0.05, log2FC > 0). (b) Heatmap of correlation analysis for differential metabolites between IBS-D and HC. Red indicates a positive correlation, and green indicates a negative correlation. (c) Bubble diagram illustrating the KEGG pathway enrichment for differential metabolites between IBS-D and HC. The abscissa represents the rich factor corresponding to each pathway, and the ordinate represents the pathway name. The size of each bubble indicates the number of metabolites enriched in the pathway. The red color gradient reflects the significance of the enrichment. (d) KEGG pathway analysis for differential metabolites between IBS-D and HC. The abscissa and the size of each bubble indicates the impact factor of the pathway in topological analysis. the ordinate and the bubble color indicates the -log10(p-value) for enrichment analysis.

Figure S3. Score scatter plot of OPLS-DA model for group IBS-D vs HC. The abscissa T score represents the predicted principal component score of the first principal component, showing differences between sample groups, the ordinate orthogonal T score represents the orthogonal principal component score, showing differences within sample groups, each scatter represents a sample, and the color of the scatter indicates different groups.

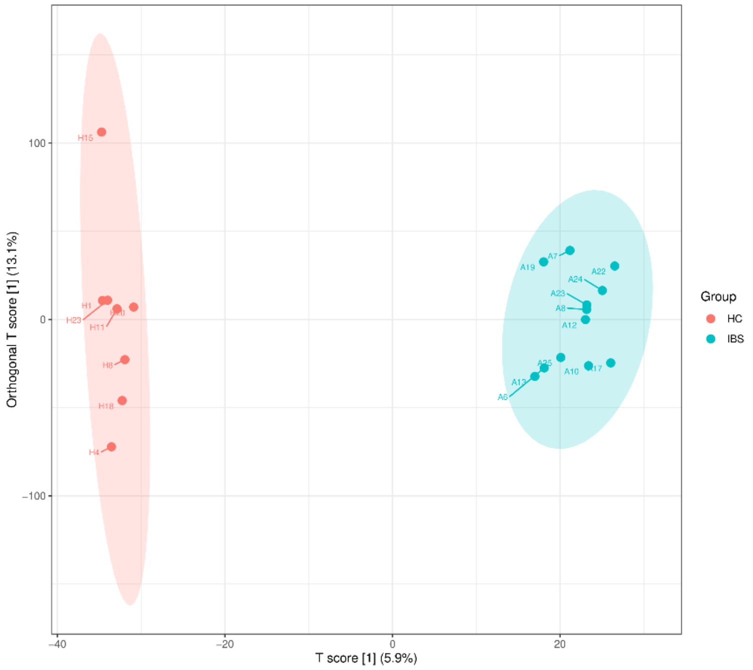

Figure S4. Power-law distributions of Single-omic co-occurrence network. Virus network: (a) and (b). Bacteria network: (c) and (d)

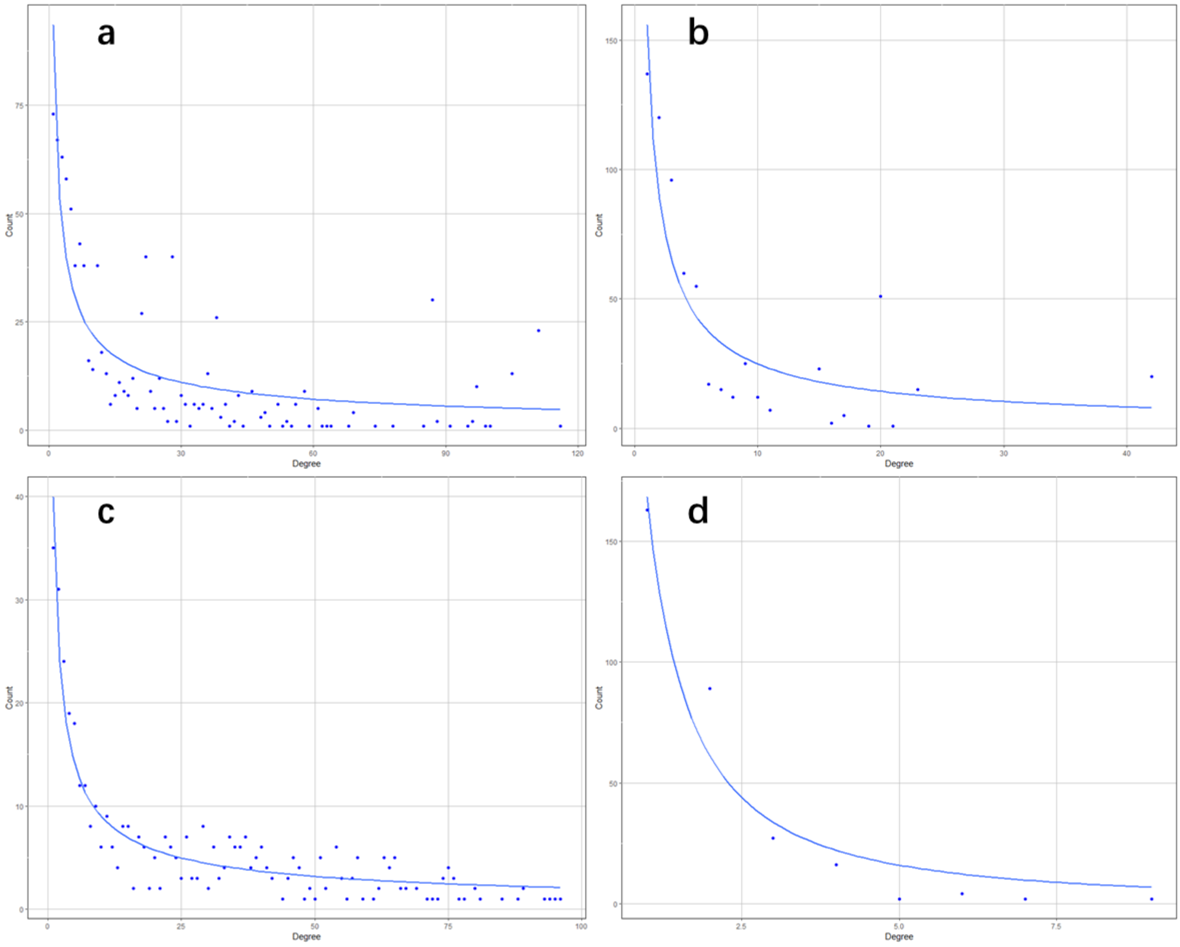

Figure S5. The Zi-Pi analyses. Virus network: (a) and (b). Bacteria network: (c) and (d).

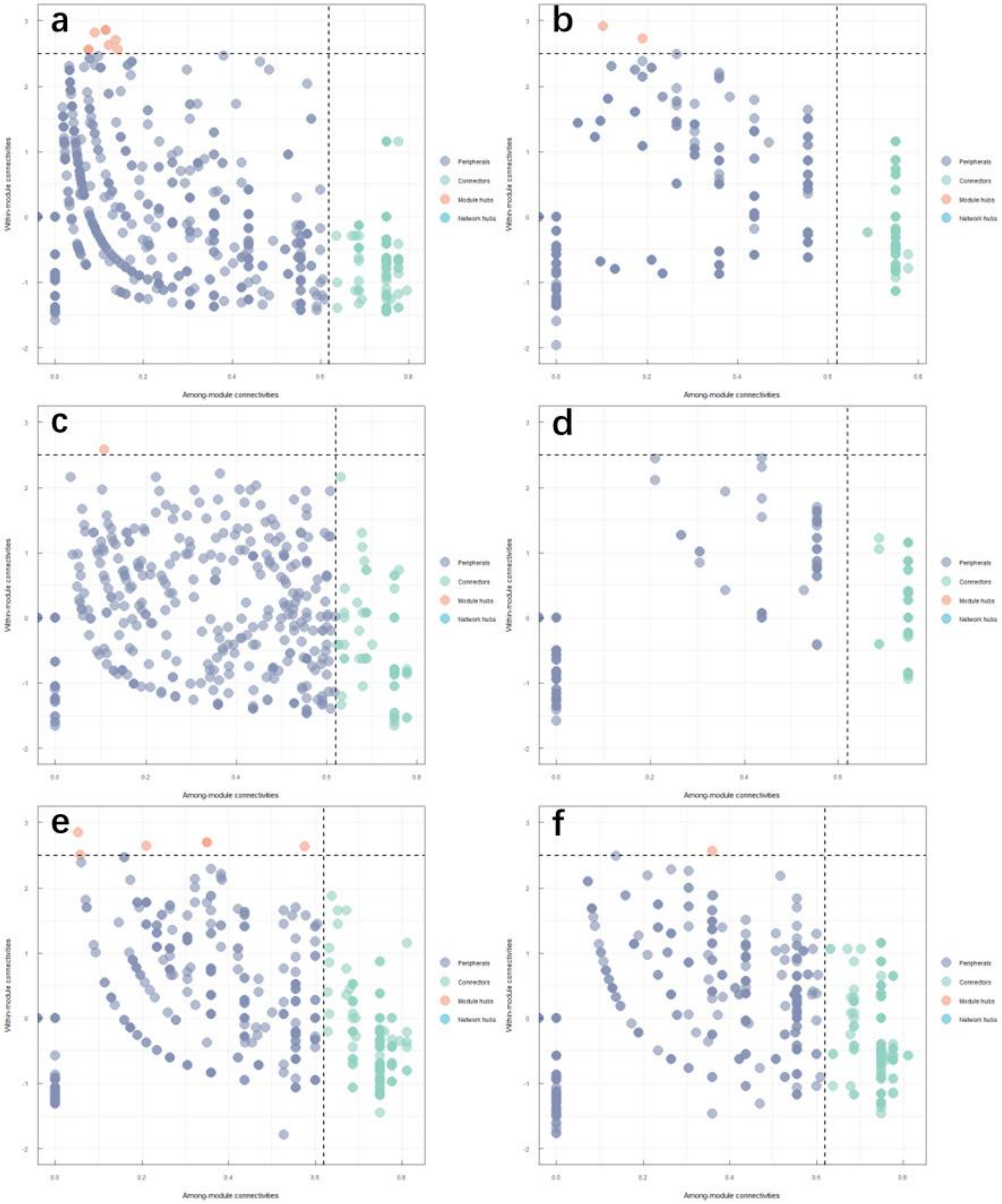

Supplement: Supplementary file 1 [file viruses-16-01054-s001.zip › viruses-3054031-supplementary.pdf]
